# Epigenetic markers in the embryonal germ cell development and spermatogenesis

**DOI:** 10.1186/s12610-022-00179-3

**Published:** 2023-02-23

**Authors:** Amadeusz Odroniec, Marta Olszewska, Maciej Kurpisz

**Affiliations:** Institute of Human Genetics, Polish Academy of Sciences, Strzeszynska 32, 60–479 Poznan, Poland

**Keywords:** Sperm epigenetics, DNA methylation, Histone methylation, Histone acetylation, Spermatogenesis, Fertilization, Spermatozoa, Epigénétique des Spermatozoïdes, Méthylation de l’ADN, Méthylation des Histones, Acétylation des Histones, Spermatogenèse, Fécondation, Spermatozoïdes

## Abstract

Spermatogenesis is the process of generation of male reproductive cells from spermatogonial stem cells in the seminiferous epithelium of the testis. During spermatogenesis, key spermatogenic events such as stem cell self-renewal and commitment to meiosis, meiotic recombination, meiotic sex chromosome inactivation, followed by cellular and chromatin remodeling of elongating spermatids occur, leading to sperm cell production. All the mentioned events are at least partially controlled by the epigenetic modifications of DNA and histones. Additionally, during embryonal development in primordial germ cells, global epigenetic reprogramming of DNA occurs. In this review, we summarized the most important epigenetic modifications in the particular stages of germ cell development, in DNA and histone proteins, starting from primordial germ cells, during embryonal development, and ending with histone-to-protamine transition during spermiogenesis.

## Résumé

La spermatogenèse est le processus de génération de cellules reproductrices mâles à partir de cellules souches spermatogoniales, dans l’épithélium séminifère du testicule. Au cours de la spermatogenèse, des événements spermatogéniques clés tels que l’auto-renouvellement des cellules souches et l’engagement dans la méiose, la recombinaison méiotique, l’inactivation méiotique du chromosome sexuel, suivis d’un remodelage cellulaire et chromatique des spermatides allongées se produisent, conduisant à la production de spermatozoïdes. Tous les événements mentionnés sont au moins partiellement contrôlés par les modifications épigénétiques de l’ADN et des histones. De plus, au cours du développement embryonnaire, une reprogrammation épigénétique globale de l’ADN se produit dans les cellules germinales primordiales. Dans cette revue, nous avons résumé les modifications épigénétiques les plus importantes dans les étapes particulières du développement des cellules germinales, dans l’ADN et les protéines histones, en partant des cellules germinales primordiales, au cours du développement embryonnaire, jusqu’à la transition histone-protamine pendant la spermiogenèse.

## Introduction

**Fig. 1:**
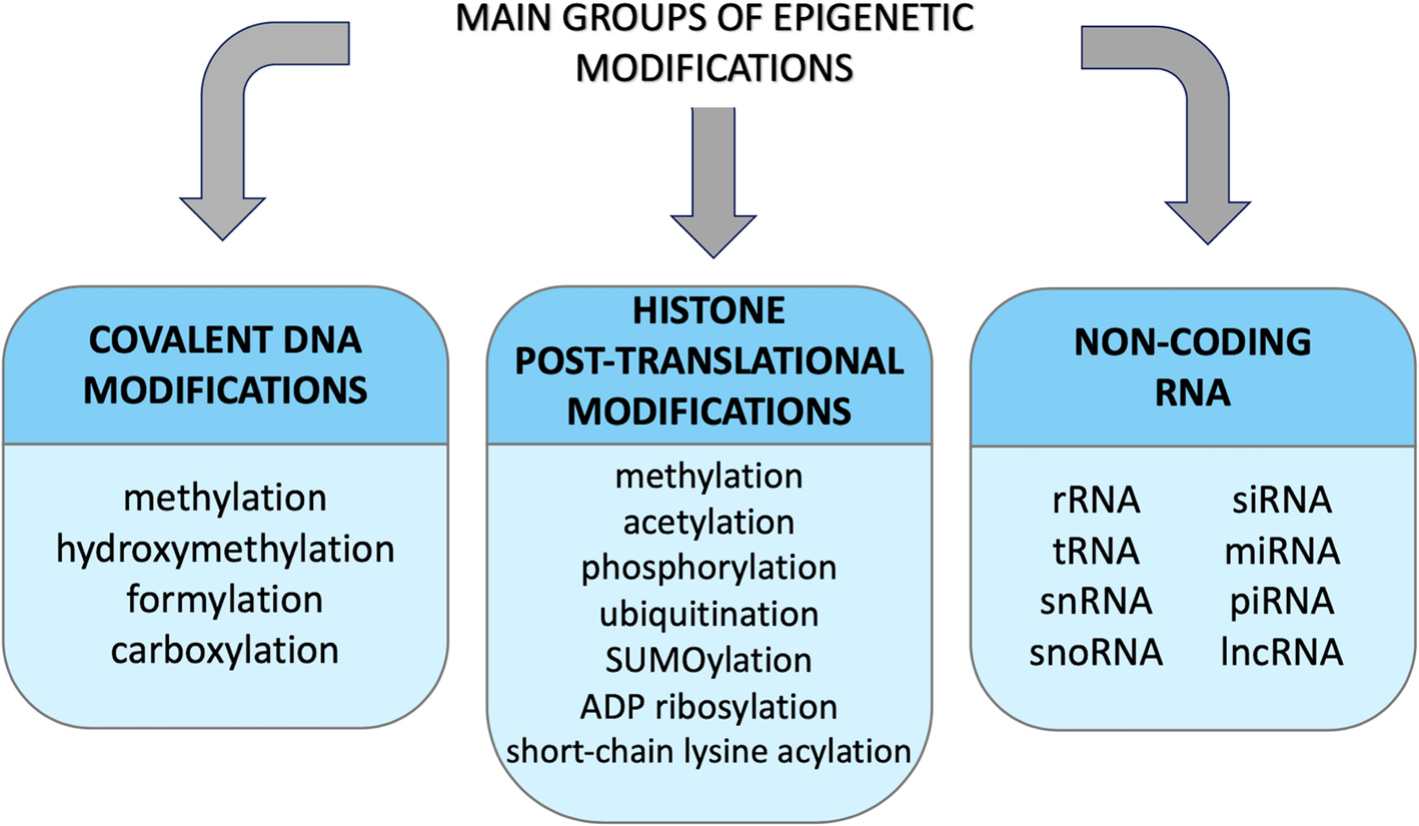
Three main groups of epigenetic modifications observed in mammalian cells. rRNA – ribosomal RNA, tRNA – transfer RNA, siRNA – silencer RNA, miRNA – micro RNA, snRNA – small nuclear RNA, snoRNA – small nucleolar RNA, piRNA – piwi-interacting RNA, lncRNA – long non-coding RNA, ADP – adenosine diphosphate, SUMO – small ubiquitin-like modifier

Epigenetics can be described as heritable alterations that do not change the DNA sequence [1–5]. In comparison to genetic changes (which affect the structure of proteins mediated by DNA mutations), epigenetic changes affect the gene expression, and in consequence protein product amount within the cell. They are reversible and associated with the response to the environment in which cells reside [1–5]. Three groups of epigenetic changes are being manifested in mammalian cells: covalent modifications of DNA bases, histone posttranslational modifications and non-coding RNA (ncRNA) (Fig. 1).

One of the first discovered and the best-known epigenetic marks to date is DNA methylation [5–7]. 5-methyl-cytosine (5mC), together with the products of its degradation: 5-hydroxymethyl-cytosine (5hmC), 5-formyl-cytosine (5fC) and 5-carboxy-cytosine (5caC), are examples of covalent modifications of DNA bases [8]. DNA methylation preferably occurs at CpG sites that are dinucleotides with cytosine followed by guanine. DNA methylation can be present at CpG sites in the intergenic region, where methylation prevents the formation of DNA mutation by silencing of the retroviral elements, or in the promotor region of the gene within the so-called CpG island (region of DNA with higher CpG density), where methylation is responsible for the control of gene expression. Additionally, methylation in the CpG island plays an important role in the paternal and maternal gene imprinting [7, 8].

Another form of DNA methylation demonstrated in the mammalian genome is non-CpG methylation. Non-CpG methylation is almost absent in adult somatic cells (0,02% of global 5mC), but the level of non-CpG methylation is elevated in embryonal stem cells, induced pluripotent stem cells, glial cells and neurons [9]. Unfortunately, the mechanisms and functions of non-CpG methylation are still not well-understood [9].

Histone posttranslational modifications are another group of epigenetic modifications. Histone modifications are being formed via the addition of a functional group to an amino acid, most commonly lysine, present within the histone tail of core histones [6]. The main role of histone modifications is the control of gene expression by chromatin condensation and decondensation [10]. Histone modifications can also provide a binding site for various proteins [11]. The already described histone modifications demonstrated in mammalian organisms are methylation, acetylation, phosphorylation, ubiquitination, SUMOylation, ADP ribosylation, short-chain lysine acylation [6, 12, 13].

The last group of epigenetic modifications is non-coding RNA (ncRNA). ncRNA is not translated into a protein and fulfills the regulatory role [14]. ncRNA can be divided into housekeeping RNAs and regulatory RNAs [15]. Housekeeping RNAs can be further divided into the following: ribosomal RNA (rRNA), transfer RNA (tRNA), small nuclear RNA (snRNA), and small nucleolar RNA (snoRNA). The housekeeping RNAs partake in the translation of mRNA into proteins and RNA processing [15]. Additionally, regulatory RNAs are involved in the regulation of gene expression toward gene silencing [14]. Regulatory RNAs can be divided into: small interfering RNA (siRNAs), microRNA (miRNAs), Piwi-interacting RNA (piRNAs), and long non-coding RNAs (lncRNAs) [14].

It is already known that roles of parental genomes are distinct after fertilization. This sex-determined diversity is being established during gametogenesis and comes out of gametic imprinting, which is a distinct methylation patterning of parental alleles determining epigenetic mechanisms in the proper development of an organism [4, 16–19]. The maternal genome is responsible for embryonic development, while the paternal one is accountable for early placental progress [4, 16–19]. Additionally, disturbances in proper methylation and demethylation rounds in gametogenic cells, followed by disruption of methylation and/or acetylation of sperm histones may lead to a lack of activation of genes crucial for normal development, resulting in occurrence of certain developmental disturbances [4, 18, 20, 21]. It is also known, that implementation of immature spermatozoa (with misaligned methylation patterns and inadequate chromatin integrity) for fertilization in assisted reproductive technologies (ART) may increase the risk of reproductive failure or offspring health status [2, 4, 22–31]. The unique epigenetic marks in sperm cells may play a key role in poising of specific gene activation in the early embryo [2, 4, 21, 25, 32]. Disturbed synchronization of the embryo genome expression may be caused by hypomethylation which may switch the process of cellular differentiation [4, 19, 33]. In this light, a cognition of the mechanisms and sense of disturbances in gametic epigenome seems to be significant, due to the relatively high frequency of ART births today (approximately 1–3% of all live births) [25, 34].

It is also documented that male infertility may be linked with changed methylation pattern in human spermatozoa, both: at the level of sperm DNA (global or in particular genes – imprinted or nonimprinted; reviewed in [35, 36]), as well as in cases with disrupted methylation in particular histones’ residues [37, 38]. Alterations in the methylation pattern were also described for males with decreased sperm chromatin integrity (disturbed protamines P1/P2 ratio), regarding to sperm apoptosis, in male ageing, in patients subjected to in vitro fertilization (IVF) procedures, in carriers of chromosomal aberrations, and in males with decreased semen quality [19, 23, 25, 39–44]. For example, in oligozoospermia where genetic causes are responsible only for 2.5–10% of observed infertility [45], the epigenetic evaluation revealed that in some patients from this group aberrant methylation patterns or imprinting errors may be causative for reduced efficiency of fertilization and elevated abortion rates [25, 33, 36, 46, 47].

Spermatogenesis is a complex process of male reproductive cells generation, that occurs within the seminiferous epithelium of male testis [48]. Spermatogenesis can be divided into several stages: spermatogonial stem cells (SSCs) self-renewal (via mitosis), differentiation of SSCs into spermatocytes, reductional division of spermatocytes into spermatids (via meiosis), and morphological transformation of spermatids into sperm cells (via spermiogenesis supported by spermiation) [48, 49]. The entire spermatogenetic process in mammals ranges from 30 to 78 days [49, 50], approximately 40 days in mice [49, 51] and 74 days in humans [52, 53]. The process of spermatogenesis is followed by the maturation of released sperm cells within the lumen of epididymis [54]. During epididymal maturation, spermatozoa acquire the ability of *zona pellucida* recognition, the acrosome reaction and progressive motility, which are necessary for proper oocyte fertilization [54]. Each step of spermatogenesis is characterized and determined by particular epimarks, both: of germ cell DNA, as well as histone residues. Taking into account the growing amount of data concerning linkage between epigenetic disturbances and reproductive problems, in this review we have been focused on summarizing the role of main epimarks crucial for the process of spermatogenesis, such as DNA methylation, and the most important histone modifications. Additionally, in a Table 1 we have collected data concerning mouse knockout models of epigenetic-related enzymes with negative effect on spermatogenesis. Thus, this review emphasizes also the epigenetic significance for reproduction besides of the strictly genetic causes, and the high number of developmental stages at which epimarking may be disturbed leading to male fertility or fertilization problems.

**Table 1:** Knockout mouse models concerning epigenetic-related enzymes with negative effect on spermatogenesis (including gene IDs from: NCBI – National Center for Biotechnology Information [55] and MGI – Mouse Genome Informatics identifying number [56])

	**Gene**	**Full name**	**NCBI/MGI number**	**Epigenetic effect**	**Knockout phenotype**	**Reference**
**Embryonic development**	*Kdm6a (Utx)*	lysine (K)-specific demethylase 6A	22,289/1095419	Persistence of H3K27me3	Disturbed PGC embryonic development due to lack of PGC marker genes expression and aberrant chromatin dynamics	[57]
	*Mthfr*	methylenetetrahydrofolate reductase	17,769/106639	Disturbed SAM synthesis	Reduced number of early germ cell due to failure of mitotic reactivation and enhanced apoptosis resulting in reduced fertility or infertility (depending on mouse model)	[58, 59]
	*Nsd1*	nuclear receptor-binding SET-domain protein 1	18,193/1276545	Reduction of H3K36me2	Absence of spermatogonia in adult testis due to aberrant de novo DNA methylation in prospermatogonia	[60]
	*Prmt7*	protein arginine N-methyltransferase 7	214,572/2384879	Loss of histone arginine monomethylation	Reduced number of PGC	[61]
	*Prmt5*	protein arginine N-methyltransferase 5	27,374/1351645	Reduction of symmetric H4R3me2	Reduced number of PGC due to lack of transposon repression during the hypomethylated DNA state	[62]
	*Setdb1*	SET domain, bifurcated 1	84,505/1934229	Reduction of H3K9me3 and H3K27me3 within endogenous retrotransposon regions	Decreased number of PGCs due to reactivation of endogenous retroviruses	[63]
**Self-renewal and commitment to meiosis**	*Dnmt3a*	DNA methyltransferase 3A	13,435/1261827	Disturbed DNA methylation	Complete spermatogenesis arrest at the spermatogonia level	[64, 65]
	*Fancd2/Fanci*	Fanconi anemia, complementation group D2 MGI:2,448,480 Fanconi anemia, complementation group I	211,651/2384790	Altered H3K9me3 and H3K4me2 in diplotene	Apoptosis of undifferentiated spermatogonia with unknown underlying mechanism, decreased number of mature sperm cells, meiotic progression not affected	[66]
	*Kdm1a*	lysine (K)-specific demethylase 1A	99,982/1196256	Increase of H3K4me2	Complete spermatogenesis arrests upon exit from the stem cell state due to persistent expression of *OCT4* gene	[67, 68]
	*Kmt2d (Mll2)*	lysine (K)-specific methyltransferase 2D	381,022/2682319	Loss of H3K4me3	Complete spermatogenesis arrests upon exit from the stem cell state due to lack of repression of *Magoh2* promotor	[69]
	*Tet1*	tet methylcytosine dioxygenase 1	52,463/1098693	Decrease of 5hmC in spermatogonial cells	Premature reproductive aging (decreased SSCs, increased apoptosis, compromised imprinting)	[70]
**Meiosis**	*Cxxc1 (Cfp1)*	CXXC finger 1	74,322/1921572	Reduction of H3K4me3	Complete meiotic arrest at the prophase I due to reduced expression of meiotic genes and reduced homologous recombination process	[71]
	*Dnmt3l*	DNA (cytosine-5-)-methyltransferase 3-like	54,427/1859287	Disturbed DNA methylation	Complete meiotic arrest at pachytene checkpoint due to loss of chromatin compaction and unpaired chromosomes	[72]
	*Ehmt2 (G9a)*	euchromatic histone lysine N-methyltransferase 2	110,147/2148922	Reduction of H3K9me1/2	Meiotic arrest at early pachytene stage due to incomplete synaptonemal complex formation	[73]
	*Hdac3*	histone deacetylase 3	15,183/1343091	Persistence of H3K9ac and H3K27ac	Spermatogenesis arrest upon exit from meiosis due to upregulation of meiosis related genes and downregulation of spermiogenesis related genes in the epigenetically independent manner (deletion of catalytic domain does not result in infertility)	[74]
	*Hr6b (Ube2b)*	ubiquitin-conjugating enzyme E2B	22,210/102944	Disturbed H2Aub	Increased apoptosis of primary spermatocytes due to synaptonemal complex aberration and increased crossing-over frequency	[75]
	*Kat8 (Mof)*	K(lysine) acetyltransferase 8	67,773/1915023	Loss of H4K16ac	Meiotic arrest due to impaired chromatin-wide expansion of γH2AX phosphorylation in leptotene and zygotene stage of meiosis	[76]
	*Kdm2b (Fbxl10)*	lysine (K)-specific demethylase 2B	30,841/1354737	Change in H3K4me3 distribution in testicular germ cells in the age-dependent manner	Gradual loss of fertility due to progressive inhibition of cell cycle	[77]
	*Paxip1 (Ptip)*	PAX interacting (with transcription-activation domain) protein 1	55,982/1890430	Reduction of H3K4me	Meiotic arrest at the zygotene stage	[78]
	*Prdm9*	PR domain containing 9	213,389/2384854	Reduction of H3K4me3 and H3K36me3 within recombination hotspots	Disturbed homologous recombination due to DSB formation impairment within recombination hotspots	[79]
	*Prmt1*	protein arginine N-methyltransferase 1	15,469/107846	Loss of asymmetric H4R3me2	Complete meiotic arrest at the zygotene-like stage due to an elevated number of DSB	[80]
	*Rnf20*	ring finger protein 20	109,331/1925927	Reduction of H2Bub	Meiotic arrest at the pachytene stage due to impaired programmed double-strand break (DSB) repair	[81]
	*Setdb1*	SET domain, bifurcated 1	84,505/1934229	Reduction of H3K9me3 within pericentromeric heterochromatin and X chromosome	Meiotic arrest at zygotene stage due to aberrant centromere clustering, bouquet formation, failure in pairing and synapsis of homologous chromosomes and MSCI failure	[82, 83]
	*Suv39h1/2*	suppressor of variegation 3–9 1/2	20,937/1099440 64,707/1890396	Reduction of H3K9me	Meiotic arrest at mid- to late pachytene stage due to delayed chromosome synapsis or incomplete pairing	[84]
	*Ubr2*	ubiquitin protein ligase E3 component n-recognin 2	224,826/1861099	Disturbed H2Aub	Increased apoptosis of primary spermatocytes due to synaptonemal complex aberration and increased crossing-over frequency	[85]
	*Uhrf1*	ubiquitin-like, containing PHD and RING finger domains, 1	18,140/1338889	Loss of H3K9me3 at the retrotransposon region and global loss of 5mC	Meiotic arrest at pachytene stage due to reactivation of retrotransposons	[86]
**Spermiogenesis**	*Camk4*	calcium/calmodulin-dependent protein kinase IV	12,326/88258	Loss of Protamine 2 phosphorylation	Spermiogenesis arrest in late elongating spermatids stage due to specific loss of protamine-2 and prolonged retention of transition protein 2	[87]
	*Cdyl*	chromodomain protein, Y chromosome-like	12,593/1339956	Reduction in histone lysine crotonylation	Reduction of male fertility with a decreased epididymal sperm count and sperm cell motility due to aberrant reactivation of sex chromosome-linked genes in round spermatids	[88]
	*Chd5*	chromodomain helicase DNA binding protein 5	269,610/3036258	Reduction of H4ac	Spermiogenesis arrest due to aberrant histone removal during histone-to-protamine replacement	[89]
	*Epc1*	enhancer of polycomb homolog 1	13,831/1278322	Reduced H4 hyperacetylation	Spermiogenesis arrest at the transition from round spermatids to elongating spermatid due to aberrant histone to protamine transition	[90]
	*JmJd1c*	jumonji domain containing 1C	108,829/1918614	Reduction of H4K16ac and HSP90 hypermethylation	Reduction of germ cells, abnormal histone-protamine remodeling and incomplete elongation of spermatids	[91]
	*Kat5 (Tip60)*	K(lysine) acetyltransferase 5	81,601/1932051	Reduced H4 hyperacetylation	Spermiogenesis arrest at the transition from round spermatids to elongating spermatid due to aberrant histone to protamine transition	[90]
	*Kdm3a (Jhmd2a/ Jmjd1a)*	lysine (K)-specific demethylase 3A MGI:	104,263/98847	Persistence of H3K9me1/2 in promoter region of Tnp1 and Prm1 genes	Spermiogenesis arrest due to incomplete chromatin condensation, extensive apoptosis and blocked spermatid elongation caused by reduced cAMP-response element modulator (CREM) regulated gene expression	[92, 93]
	*Parp1/2*	poly (ADP-ribose) polymerase family, member 1/2	11,545/1340806 11,546/1341112	Abnormal ADP ribosylation of DNA damage response proteins	Reduced fertility caused by abnormal sperm morphology due to abnormal histone retention	[94]
	*Pygo2*	pygopus 2	68,911/1916161	Reduction of H3K9/14ac	Spermiogenesis failure due to reduced expression of Prm1, Prm2, Tnp2 and H1fnt	[95]
	*Rnf8*	ring finger protein 8	58,230/1929069	Reduction of H2Aub and H2Bub	Spermiogenesis arrest at late spermatid stage due to aberrant histone removal during histone-to-protamine replacement	[96]
	*Scml2*	Scm polycomb group protein like 2	107,815/1340042	Persistence of H2AK119ub on sex chromosome	Disturbed reactivation of escape genes located on sex chromosome during spermiogenesis	[97]
	*Setd2*	SET domain containing 2	235,626/1918177	Loss of H3K36me3	Complete spermatogenesis arrests at round-spermatid stage due to acrosomal malformation and histone-to-protamine replacement malfunction	[98]
	*Sirt1*	sirtuin 1	93,759/2135607	Reduction of H4 hyperacetylation	Reduced fertility due to defects in the histone to protamine transition	[99]
	*Sly* *Dot1l*	Sycp3 like Y-linked DOT1-like, histone H3 methyltransferase	382,301/2687328 208,266/2143886	Reduction of H3K79me2 and H4 hyperacetylation	Impaired histone to protamine transition due to aberrant chromatin structure	[100]
	*Tssk6*	testis-specific serine kinase 6	83,984/2148775	Loss of γH2AX phosphorylation	Spermiogenesis arrest due to retention of histone H3 and H4 during histone-to-protamine transition	[12]

## Major epigenetic modifications of DNA

### Methylation (5mC)

DNA methylation is a result of a transfer of a methyl group from S-adenosyl-L-methionine (SAM) to the fifth carbon atom of the cytosine residue [101]. This reaction is performed by a group of specialized enzymes called DNA methyltransferases (DNMTs). In mammals, three of them coordinate DNA methylation: DNMT1 (DNA methyltransferase 1), DNMT3a (DNA methyltransferase 3 alpha), and DNMT3b (DNA methyltransferase 3 beta) [102]. During spermatogenesis, two distinct types of methylation occur: de novo methylation, and so-called maintenance methylation [103]. The first of them is performed by DNMT3a and DNMT3b and allows the methylation of previously unmethylated DNA regions [103]. The latter uses the DNMT1 enzyme to sustain DNA methylation after meiotic division. DNMT1 has an affinity to hemi-methylated DNA in the replication fork [103]. DNMT3L (DNA methyltransferase 3 like) is the next methyltransferase present in mammals, but it lacks a catalytic domain, and therefore has no catalytic activity [101]. DNMT3L recognizes the unmethylated region of histone H3 and activates DNMT3a and DNMT3b methyltransferases by acceleration of DNA and S-adenosyl-L-methionine (SAM) binding to methyltransferases [104]. Passive or active demethylation is further responsible for removal of DNA methylation. Passive demethylation is coupled with cell division and lack of methylation maintenance, while active demethylation is associated with the oxidation of methyl groups to hydroxymethyl group [105, 106].

The role of DNA methylation depends on its location within the DNA structure. In the intergenic region, bulk DNA methylation prevents the expression of potentially harmful genetic elements like incorporated retroviral genetic material [8]. Methylation within CpG island shores regulates tissue-specific gene expression, while methylation in CpG islands contributes to genomic imprinting by the stable silencing of one copy of the gene [8]. The level of DNA methylation remains roughly constant throughout the life of an individual [107]. The highest level of DNA methylation was detected in the brain and thymus, while the lowest levels were recorded in mature sperm cells and placenta [107], but the difference is negligible [108].

### Hydroxymethylation (5hmC)

DNA hydroxymethylation is a well-described intermediate product of active demethylation. Ten-Eleven Translocation (TET) family proteins can oxidize the 5mC to 5-hydroxymethylcytosine (5hmC) [106, 109–112]. The same enzymes can further oxidize 5hmC to 5-formylcytosine and 5-carboxylcytosine, from which the carboxyl group is removed by thymine-DNA glycosylase (TDG) coupled with base excision repair (BER) mechanism to restore unmethylated cytosine [106, 109, 111, 112]. The level of DNA hydroxymethylation is tissue-specific and has been mainly demonstrated within regulatory elements of the gene [113, 114]. 5hmC level has been observed as the highest in brain tissue [108]. Also, in primordial germ cells (PGCs) the 5hmc level is observed at a high level [115]. In embryonal cells, 5hmC level originates out of genome-wide demethylation, which leads to epigenetic reset and the 5mC landscape restructuring during the development of specific tissues [113, 115]. Additionally, hydroxymethylation presence is associated with transcription of tissue-specific genes [113, 114], which can be especially important in constantly changing organs that must adapt to environmental signals (such as the brain), where the 5hmc level is the highest among the tissues. In sperm cells, 5hmC level is four times lower than that in somatic cells [116].

## Major epigenetic modifications of histones

### Methylation

Histone methylation is a modification resulting from the transfer of the methyl group from SAM to ε-amino group of lysine (K) or ω-guanidino group of arginine (R) residues, mostly on the N-terminal tail of histones H3 or H4 [117]. The lysine residue can be mono-, di- or tri-methylated, while an arginine residue can be mono- or di-methylated [117]. Methylation does not change the charge potential of modified histone and can be associated either with activation or repression of gene expression [10]. Histone methyltransferases (HMTs) are the catalyzers for the methylation of histones [10]. HMTs that add a methyl group to the lysine residue are called lysine methyltransferases (HKMTs), while the methylation of arginine residue is performed by protein arginine methyltransferases (PRMTs) [118]. Most lysine methyltransferases contain the SET (Su(var)3–9, Enhancer-of-zeste and Trithorax) domain as their catalytic domain [119, 120]. However, the DOT1L (disruptor of telomeric silencing-1-like) is a unique lysine methyltransferase that lacks a SET domain and catalyzes the methylation of only lysine 79 residue of histone H3 (H3K79) in the histone core [120]. HMTs methylate their substrate to a defined level and specific changes in the catalytic site of the amino acid sequence can alter the level of methylation activity [121]. For example, the mutation F281Y in *Neurospora crassa* defective in methylation-5 (*Dim5*) histone H3 lysine 9 methyltransferase gene can change the enzyme activity from trimethylase to monomethylase [121]. A similar effect is observed for the equivalent mutation F1205Y in human *Dim5* homolog euchromatic histone-lysine N-methyltransferase 2 gene (*G9A*), where this mutation changes activity from demethylase to monomethylase [121]. Histone demethylation is performed by histone demethylases (HDMTs) [120]. Demethylation can also be performed using protein-arginine deiminase type-4 (PADI4) enzyme in the process of deamination of monomethylated arginine residue to citrulline without arginine regeneration [122].

For example, the abbreviated form of methylation description is H3R8me2 for dimethylation of arginine 8 residue in histone H3, and H3K36me3 for trimethylation of lysine 36 residue in histone H3.

### Acetylation

Histone acetylation is a modification resulting from the transfer of the acetyl group from acetyl-CoA to ε-amino group of the lysine side chains in N-terminal tail of core histones (H2A, H2B, H3, H4) [117]. Acetylation neutralizes the positive charge potential from lysine residues, consequently weakening the interaction between DNA and histones. Those changes cause loosening of chromatin and lead to transcriptional activity [123]. Additionally, histone acetylation regulates protein–protein interaction via bromodomains and in consequence takes part in histone deposition and DNA repair [123]. Acetylation is performed by histone acetyltransferases (HATs) [124]. HATs are classified as type A or type B transferases. Enzymes included in type A are localized in the cell nucleus and contain bromodomains, which allow them to bind and acetylate histones already embedded in chromatin structure [124]. However, type B acetyltransferases are located in the cytoplasm and can modify only newly synthesized histones. All type B enzymes mainly acetylate newly synthesized histones in the cytoplasm and are more conservative [124]. Deacetylation is performed using histone deacetylases (HDACs). They are less site-specific when compared to HATs, and commonly create large complexes with each other and additional proteins [125].

For example, the abbreviated form of acetylation description is H4K5ac for the lysine 5 residue in histone H4.

### Phosphorylation

Histone phosphorylation is a modification resulting from the transfer of the phosphate group (PO_4_) from ATP to the hydroxyl group of serine, threonine, tyrosine side chain, mainly in the N-terminal tail of histones [117]. Phosphorylation of these amino acids introduces an additional negative charge potential to the histone, which then changes chromatin structure [117]. A phosphate group presence increases the ability for DNA binding by transcriptional factors and enzymes. Next, the attached enzymes can add new post-transcriptional modifications (PTMs) or participate in double-strand breaks (DSBs) repair [126]. The phosphate group can be attached to the histone tails by kinases and detached by phosphatases [117].

For example, the abbreviated description of phosphorylation is H3S10ph for serine 10 residue in histone H3.

### Ubiquitination

Histone ubiquitination is a process of an addition of a small 76 amino acid protein called ubiquitin mainly to the ε-amino group of lysine residue in a side chain via the covalent isopeptide bond [127]. Ubiquitination is catalyzed by a sequence of 3 enzymes: ubiquitin-activating enzyme (E1), ubiquitin-conjugating enzyme (E2) and ubiquitin ligase (E3) [128]. Poly-ubiquitination is created by the extension of mono-ubiquitination at lysine residues: K6, K11, K27, K29, K33, K48, and K63 or methionine M1 [129]. The function of ubiquitination depends on the location within the histone tail, and whether a histone is mono- or poly-ubiquitinated. Mono-ubiquitination primarily controls gene expression probably via the changing chromatin structure or by providing an interaction surface for other protein complexes [130], while poly-ubiquitination is involved in a wide range of processes, including protein to protein interaction (i.e., K63-linked ubiquitin chains in DSB repair response), or protein guidance towards degradation in proteasomes (i.e., K48-linked ubiquitin chains) [131]. Ubiquitination is removed by deubiquitinating enzymes (DUBs) [128].

For example, the abbreviated description of ubiquitination is H2BK120ub for lysine 120 residue in histone H2B.

### Other histone modifications

Of the described modifications, the less common modifications can also be present on histone tails, i.e., SUMOylation (a modification by small ubiquitin-like modifiers), a group of short chain lysine acylations including: crotonylation, or butyrylation(s), (widely reviewed in [132]), or PARsylation poly(ADP-ribose) metabolism. However, there is only little literature data available concerning their role, so far [133, 134], thus we made only a brief description here. It was already shown that SUMOylation represents a marker of defective sperm quality (motility and morphology), is linked to chromatin remodeling and constitutive heterochromatin [135–139]. In *D. melanogaster* a general lysine crotonylation depends on the acetylation status of the spermatid chromatin, while in mice crotonylation was strictly linked to transcription regulation of sex chromosome-linked genes in round spermatids and genome-wide histone replacement in elongating spermatids [88, 140, 141]. Additioanlly, dysregulation of crotonylation by Cdyl (chromodomain Y-like transcription corepressor) resulted in a lower sperm count and motility [88]. A role of other modifications from butyrylation subgroup, have been linked to active gene transcription in meiotic and post-meiotic cells in male germ cells, [142]. PARsylation has been found in elongating spermatids (mouse study) as an important player for proper sperm nucleoprotein exchange and correct sperm head formation [143, 144].

## Epi-changes in primordial germ cells

Spermatogenesis is a complex process that starts from spermatogonial stem cells (SSCs) and ends with mature spermatozoa [50]. Primordial Germ Cells (PGCs), which are the more primitive ancestors of SSCs, are at least equally important for sperm production from an epigenetic standpoint [145]. At this stage, major DNA methylation modifications occur, which ensure appropriate epigenetic patterns in developing gonocytes [145, 146]. The pluripotency of PGCs and their open chromatin state give rise to SSCs by undergoing asymmetrical cell divisions [145, 146]. There are also suggestions (but still disputable), that PGCs have been reported to survive in low numbers in multiple adult tissues (including testes, both mice and human) as VSELs (very small embryonic like stem cells), that are epiblast-derived cells deposited during early gastrulation playing an important role in turnover of tissue specific/committed stem cells, and expressing several markers characteristic for pluripotent stem cells [147–150]. The pluripotent VSELs express variety of PGCs markers, and their number is increased in testicular pathologies (i.e., cancer), revealing reduced 5mC expression and altered *Igf2-H19* (H19 imprinted maternally expressed transcript – insulin like growth factor 2) pattern and thus influencing the epigenetic profile [147–150].

**Fig. 2:**
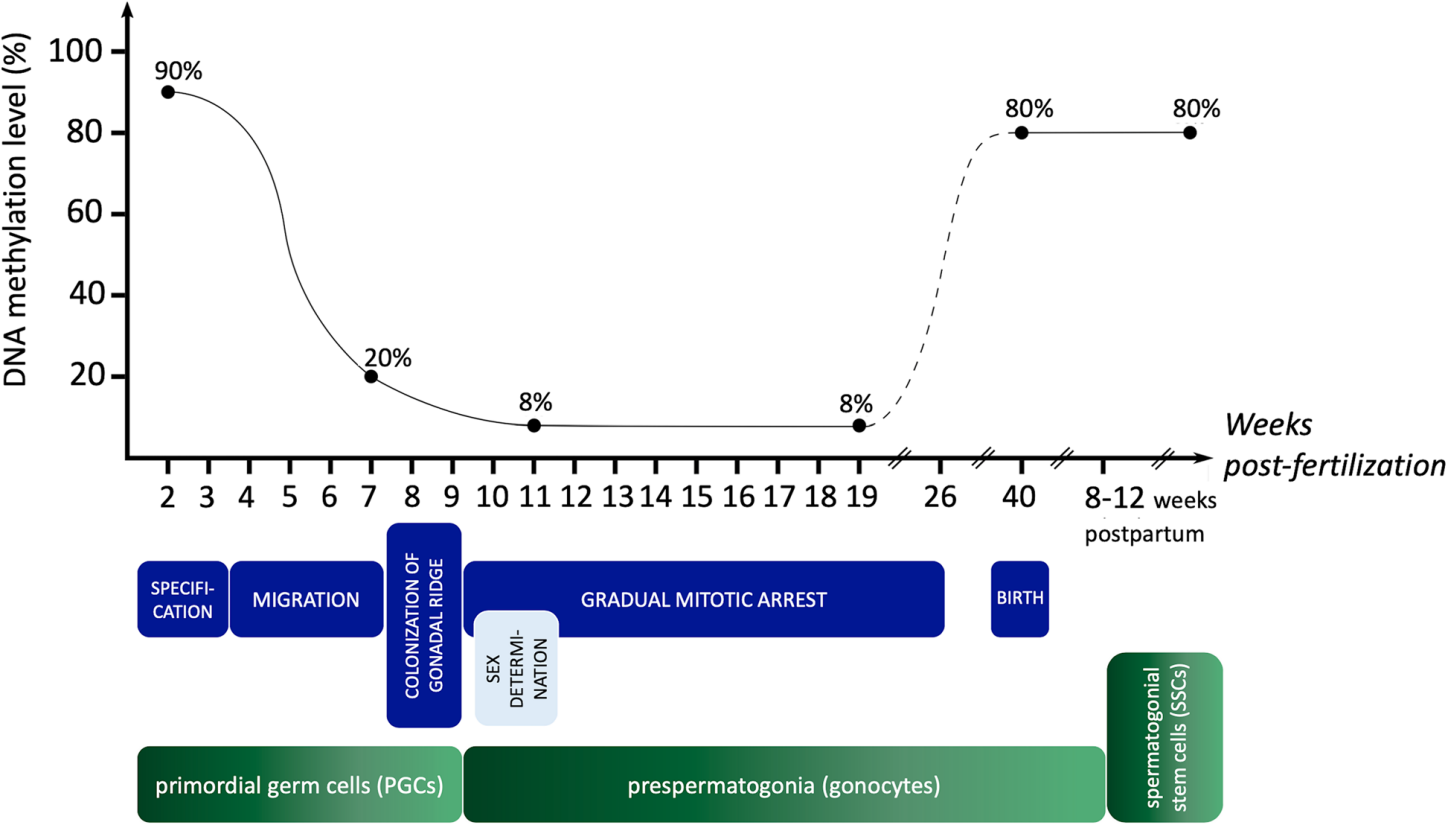
Global DNA methylation changes during human embryonal development. Dashed line means the estimated (but still unknown) time points of the remethylation phase in arrested gonocytes. The full methylation pattern is established either until birth or before puberty, and is sustained to the end of spermatogenesis. Dots represent the most important steps of DNA methylation. PGCs – primordial germ cells, SSCs – spermatogonial stem cells

Three stages can be listed during PGC development: specification, migration, and colonization [151]. They are timed, as follows: embryonic day E6.0-E8.0, E8.0-E10.0, and E10.0-E13.0 in mice, while in humans: 2.0–3.0 weeks, 5.5–8.0 weeks, and 8.5–9.0 weeks of embryonal development [146, 151]. PGCs undergo massive DNA demethylation, mainly in a replication-coupled manner. This change is associated with rapid cell cycle progress, the lack of a specific gene expression (i.e., developmental pluripotency-associated protein 3 gene – *STELLA,* ubiquitin like with PHD and ring finger domains 1 gene—*UHRF1*), and repression of DNMT3a, and DNMT3b activity [102]. This results in the absence of both maintenance and de novo methylation. Demethylation is essential for the erasure of genomic imprints, which could affect the particular stages of spermatogenesis [102]. The wave of demethylation in mice happens between E6.5-E10.5 and E10.5-E12.5 and includes approximately 90% of genome-wide loss of 5mC [146], while in humans starts before 7 weeks of embryonal development, with global methylation decreasing from more than 80% to approximately 20% (Fig. 2) [145, 152]. In the 11-week human embryos, DNA methylation reaches its lowest level at approximately 8%. This status is maintained until at least until week 19^th^, which indicates that de novo methylation occurs later in embryo development [145]. More importantly, the differentially methylated regions (DMRs) of imprinted genes are completely demethylated from week 10^th^ at least until the week 19^th^ [145]. However, some regions in DNA retain relatively substantial methylation, namely the alpha satellite regions in the centromeric and pericentromeric regions of the chromosomes (36.5% of DNA methylation retained), and evolutionary young and active transposable elements with 24% of methylated DNA [145]. Also, hydroxymethylation of DNA was observed in PGCs in male 10-week embryos at a low level of approximately 2%. The presence of 5hmC indicates an auxiliary role of the active process in global DNA demethylation [145].

After PGCs colonize gonads, they undergo a sex-specific transformation toward gonocytes, and between the weeks 9–26 they gradually enter the mitotic arrest for the remaining phase of fetal development [153, 154]. Mitotic divisions resume 8–12 weeks after birth, and gonocytes transform into self-renewing spermatogonial stem cells (SSCs) [155]. De novo methylation starts to emerge in mitotically arrested gonocytes [156]. The global methylation pattern is established before birth [157], while full methylation of paternal imprints (*H19-IGF2* and *DLK1-DIO3*: delta like non-canonical Notch ligand 1 – iodothyronine deiodinase 3) is fully established only before the onset of meiosis during puberty [156]. Simultaneously, maternally imprinted genes remain fully unmethylated from the PGC stage onwards [156]. Also, the expression of DNA methyltransferases changes in time. All the three of them follow a similar expression pattern. In mice, high levels of DNMT1, DNMT3a, and DNMT3b are being observed in spermatogonium A, leptotene/zygotene spermatocytes, and round spermatids [101].

**Fig. 3:**
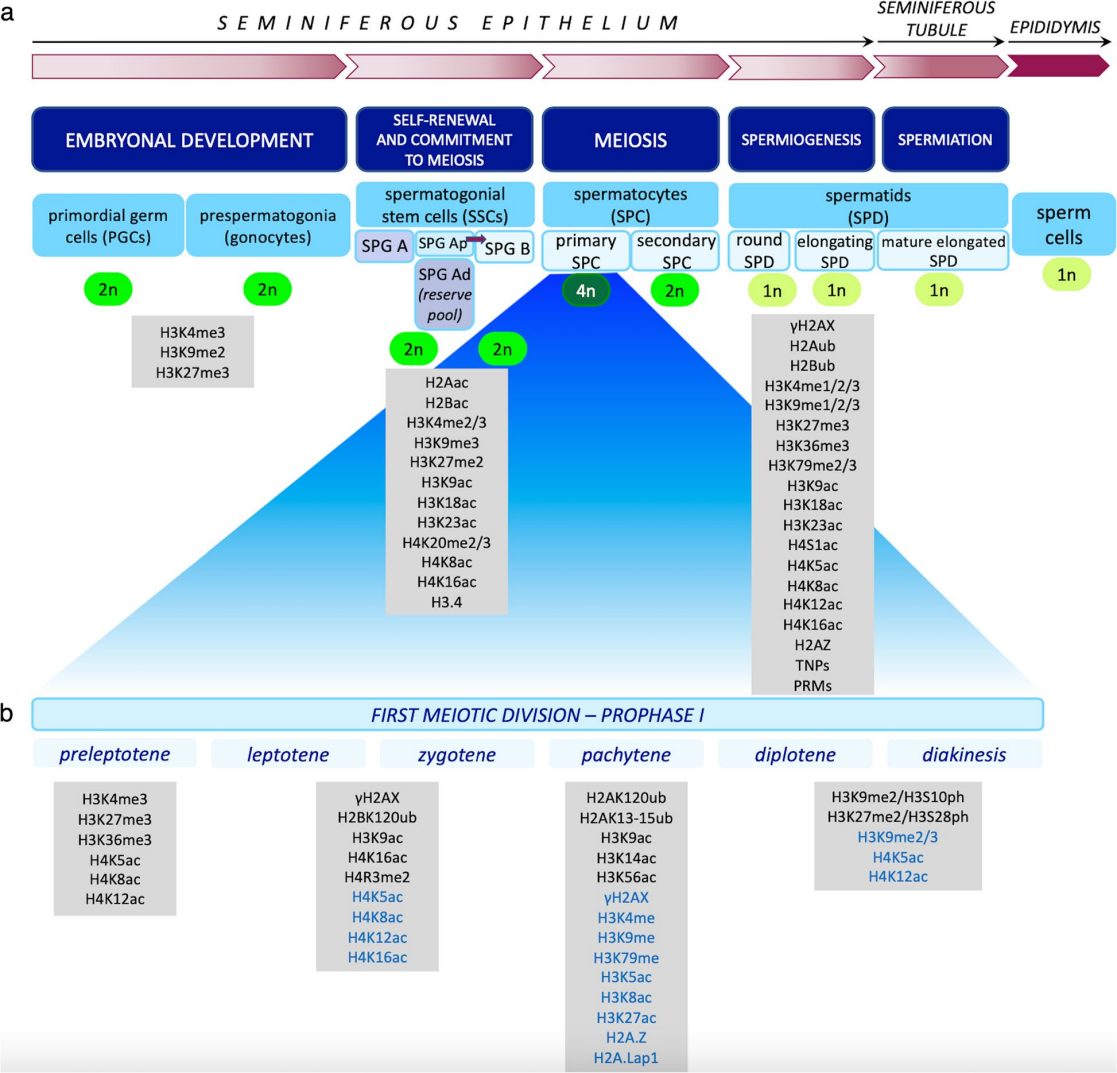
The most important histone post-translational modifications at particular stages of human spermatogenesis. **a** The main histone post-translational modifications during embryonal development, mitotic proliferation, and spermiogenesis, together with indication of the cell type, their ploidy and morphology. **b** The main histone post-translational modifications during prophase I (the most important stage of first meiotic division in primary spermatocytes). Epigenetic modifications found on autosomal chromosomes are marked in black, while modifications representative for sex chromosomes are marked in blue. PGCs – primordial germ cells, SSCs – spermatogonial stem cells, SPC – spermatocyte, SPD – spermatid, ac – acetylation, me – methylation, ph – phosphorylation, ub – ubiquitination, 1n – haploid genome, 2n – diploid genome, 4n – tetraploid genome

PGCs also exhibited dynamic changes in histone modification patterns (Fig. 3). Three main modifications occur at lysine 4, 9 and 27 residues (K7, K9, K27) of histone H3. Both H3K4me3 (activating) and H3K27me3 (repressive) are present in the promoter region of somatic genes (i.e., *Hox*: Homeobox proteins gene*, Gfap*: Glial fibrillary acidic protein gene) as a bivalent histone modification and they ensure gene repression in an absence of DNA methylation [37, 158]. However, in the promoter region of PGC-specific genes only activating H3K4me3 modification was observed, which corresponds to an active gene expression [158]. Additionally, bivalent marks are distributed differently in female and male PGCs, suggesting their role in establishing male and female-specific methylation patterns [159]. Furthermore, H3K4me3 is expected to be involved in the blocking of the de novo DNA methylation, as Dnmt3L binds only to unmethylated H3K4 [104, 160]. In PGCs the global reduction of H3K9me2 and increase in repressive H3K27me3 are being observed in mice at E8.5, while in humans after week 9 of gestation, and these changes are likely associated with the activation of PGC-specific genes, such as *Ddx4* (DEAD-box helicase 4), *Dazl* (deleted in azoospermia-like), and *Stra8* (stimulated by retinoic acid gene 8) [161–163]. Simultaneously, H3K9 acetylation increases, while H3K9 trimethylation remains at a high level [162].

## Self-Renewal of spermatogonia and commitment to meiosis

**Fig. 4:**
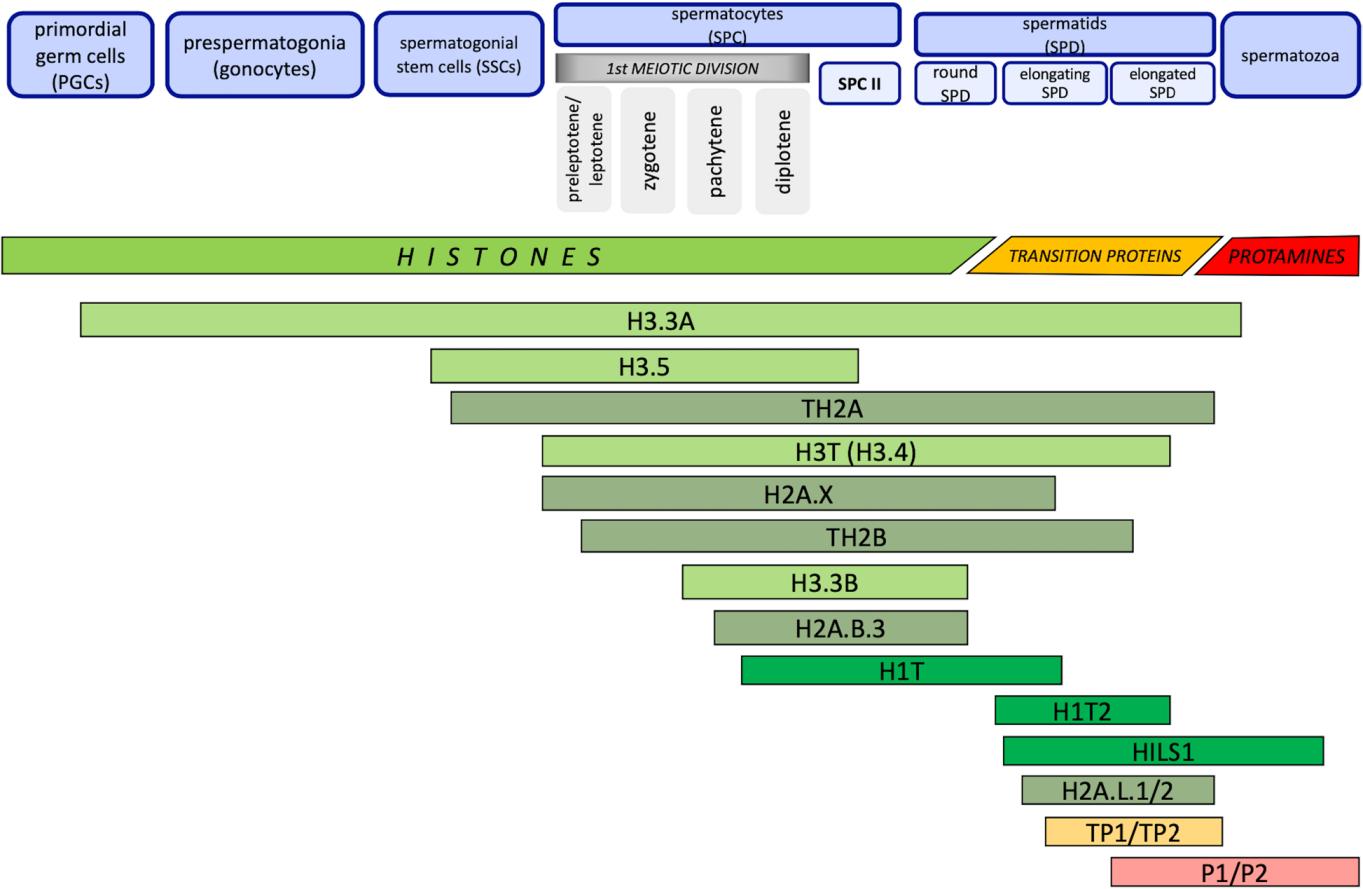
Main variants of the histones during the spermatogenetic process (human and mice)

Spermatogenesis starts with spermatogonial stem cells (SSCs), also known as ‘spermatogonia A type’ (SPG A), ready for replication via mitosis [102]. This process enables the cells to multiply continuously and maintain a constant number of stem cell reservoir within the gonad. Two types of SPG A in human testes can be found: SPG A dark (SPG Ad), and SPG A pale (SPG Ap). The first one is associated with the self-renewal of SSCs and constitutes a reserve pool of gonadal stem cells [164]. Pale spermatogonia A can also be subjected to mitotic self-renewal divisions, but in addition, they can differentiate into spermatogonia B (SPG B) [164]. In the next step, SPG B transform into primary spermatocytes, which enter the first meiotic division. Those transitions are controlled by the activation and repression of specific genes by post-translational modifications of histone tails [117]. Acetylation of histone H4 (H4K8, H4K16) prefers the neighbourhood of transcription start sites (TSSs) in SSCs, but it controls only constitutively active genes [165]. Also, the acetylation of histone H3 (i.e., H3K9, H3K18, H3K23), and variants: H2A, and H2B is present in both SPG A and SPG B cells [166]. Those modifications might be necessary during DNA replication [117]. Histone methylation fulfils the critical role in maintaining the balance between self-renewal of spermatogonia and commitment to meiosis. Commonly occurring histone methylation sites are H3K27, H4K20 in their dimethylated states, and H3K9, H4K20 in trimethylated states [167]. Those PTMs interact with promyelocytic leukaemia zinc finger transcriptional repressor (PLZF), which is essential in the maintenance of the stem cell pool [167]. But the most important modification responsible for the stem cell persistence in a pluripotent state is the methylation of lysine 4 residue in histone H3 (H3K4me2/me3) [37, 38, 67]. This modification can exist in constitutive heterochromatin (in the dimethylated state) or facultative heterochromatin (in the trimethylated state). It is present at the promoter and enhancer regions of genes associated with pluripotency (i.e., octamer-binding transcription factor 4—*OCT4,* Nanog homeobox – *NANOG*) and activates their transcription [38, 67]. H3K9me works oppositely to H3K4me and is added by histone lysine methyltransferase G9a in a promoter region of the same genes. However, it acts through the blocking gene transcription and – in consequence – promoting cell differentiation [73]. Demethylation of H3K9me restores self-renewal phenotype in SSCs [117]. After the commitment to meiosis, SSCs undergo numerous changes in histone profile, among which the most prominent ones are modifications in H3 and H4 methylation profiles, and incorporation of a new variant of histone H3 called H3.4 in human, and H3t in mice [168–170]. The changes in methylation pattern mainly occur in the following: H3K4, H3K9, H3K27, and H4K20. In early spermatogonia, repressive methylations like H3K9 and H3K27 occur. Their role is to impede genes’ action associated with pluripotency, such as *NANOG* (Nanog homeobox), *SOX2* (SRY-box transcription factor 2), *LEFTY* (left–right determination factor), and *PRMD14* (PR/SET domain 14) [117]. In late spermatogonia, transcriptionally activating modifications as H3K4me strengthen, and are probably linked with an increase in the expression of genes necessary at the early stages of meiotic division [118]. The histone variant H3t in mice (H3.4 in human) replaces canonical H3 in SPGs B and early spermatocytes. H3t is probably introduced during mitotic divisions throughout the entire genome, except for sex chromosomes [168, 169]. The incorporation of H3t into chromatin structure causes its decondensation due to more flexible entry-exit regions of H3t compared to canonical H3 histone [168, 169]. The absence of histone H3t in mice results in a suspension of SPG differentiation into spermatocytes [168, 169]. An interesting – but still scantly described – is variant H3.5 observed in testes of patients with normal spermatogenesis in spermatogonia or preleptotene/leptotene-stage primary spermatocytes, where accumulated around TSS sites may play a role in the chromatin loosening [171, 172]. The general representation of histone variants during particular stages of spermatogenesis has been summarized in Fig. 4 and detailed reviews [169, 170].

## Meiosis

The next step of spermatogenesis is meiosis – a complex process with the main goal to reduce cell ploidy from 2n (diploid) to 1n (haploid) [173]. One tetraploid (4n) primary spermatocyte enters meiosis, and after two meiotic divisions results in four haploid spermatids [174]. Spermatogenesis consists of two consecutive divisions with the intermediate product in the form of diploid secondary spermatocytes [174]. During spermatogenesis, the most important point is the first phase of meiosis I called prophase I. Dividing cells spend overwhelming majority of meiosis time in prophase I, the phase full of actions crucial for proper cell division [173, 175]. The first one is meiotic recombination, known as the crossing-over [176]. This process is critical for the maintenance of genetic diversity in the offspring. Lack of crossing-over events leads to disturbances in maintenance of proper ploidy in gametes, and then to infertility problems. In some regions of DNA, so-called: ‘hot spots’, recombination occurs more often [176]. The other important process is meiotic sex chromosome inactivation (MSCI). The absence of MSCI induces meiotic arrest at the pachytene stage of prophase I due to numerous unrepaired DNA double-strand breaks (DSB) [177]. The third one crucial for prophase I is the synapsis of homologous chromosomes, which is necessary for later phases of meiosis I. It also allows mechanisms mentioned above to be carried out correctly [176, 177]. Also during prophase I, great changes in the expression of meiotically associated genes are being observed, such as: *TEX19* (testis expressed 19), *PRDM9* (PR/SET domain 9), *SYCP3* (synaptonemal complex protein 3), and *BRDT* (bromodomain testis associated) [126, 178]. All those events are completely or partially controlled by histone epigenetic modifications. Prophase I can be divided into 5 major stages: leptotene, zygotene, pachytene, diplotene and diakinesis.

### Preleptotene

During the preleptotene stage of prophase I, preparation for meiotic recombination of primary spermatocyte begins. In this stage of prophase I, chromosomes are loosely packed and appear singly [179]. To keep the chromatin stable in a loose state, mostly the histone modifications with decondensing type of influence are present, mainly acetylation of histone H4 lysine residues 5, 8, and 12 (H4K5ac, H4K8ac, H4K12ac) [180]. In both: human and mice, the main role of H4 acetylation is to unlock access to recombination sites, creating recombination hot spots by DNA-binding zinc-finger protein (PRDM9) and trimethylation of histone H3 on lysine 4 residue (H3K4me3) [181, 182]. This modification is the most important mark of recombination hot spots, and it is used for further loosening of the chromatin structure [182]. In mice, Prdm9 can also trimetylate lysine 36 residue (H3K36me3), and then leads to the creation of the bivalent signature exclusively in the region of recombination [182]. Repressive histone marks are mostly absent in the recombination hot spots, in the exception of the coincidence of H3K27me3 and H3K4me3 in bivalent recombination sites [183].

### Leptotene and Zygotene

At the leptotene stage, the first step of homologous recombination (HR) occurs. In both: human and mice, topoisomerase Spo11 induces double-strand breaks (DSBs) within hot spots of relaxed DNA chromatin [176]. Two copies of the Spo11 enzyme create an asymmetric DNA break, while each copy cuts one strand of DNA [176, 184]. After the appearance of DSB, the most important modification for this stage – the phosphorylation in serine 139 residue of the histone variant H2AX is added (also known as γH2AX, expressed especially at preleptotene, but weakly also in spermatogonial cells, primary pachytene spermatocytes and in elongated spermatids) [126, 185]. Three waves of phosphorylation of the γH2AX have been observed during prophase I, two of which are related to HR [76]. The first wave occurs during leptotene and is performed by ATM serine/threonine kinase, while the second one depends on ATR serine/threonine-protein kinase and is present from the early to mid-zygotene stage [76]. Ubiquitination of lysine 120 residue on histone H2B (H2BK120ub) by ring finger protein 20 (RNF20) is another active modification during the zygotene [186]. Both γH2AX and H2BK120ub are responsible for loosening of chromatin structure and recruitment of repair proteins like: the mediator of DNA damage checkpoint protein 1 (MDC1), Mre11, Rad50, and Nbs1 (MRN) complex, and breast cancer type 1 susceptibility protein (BRCA1) and this process is similar in human and mouse [117, 186]. For correct phosphorylation and ubiquitination, proper methylation and acetylation of histone H4 must be established. Especially, the dimethylation of arginine 3 residue in histone H4 (H4R3me2) performed by protein arginine methyltransferase 5 (Prmt5) [187], and acetylation of lysine 16 residue in histone H4 (H4K16ac) by MOF (male absent on the first) histone acetyltransferase [76]. The absence of Prmt5 and MOF results in a lack of proper epigenetic marking, leading to inappropriate γH2AX phosphorylation as a consequence [76, 187].

During the leptotene and zygotene stages of the prophase, the acetylation of lysine 9 residue in histone H3 (H3K9ac), and lysine 5, 8, 12, 16 residues in histone H4 (H4K5ac, H4K8ac, H4K12ac, H4K16ac) occurs in X and Y chromosomes [188]. Those modifications resemble transcriptionally active chromatin and might be associated with the activation of genes located within sex chromosomes [188]. Correspondent modifications, namely H4K5ac, H4K8ac, and H4K12ac, can also be found in autosomal chromosomes where they play a similar role [180].

### Pachytene

During the pachytene stage of prophase, DSBs induced in leptotene are repaired using Holliday junctions (HJs) or synthesis-dependent strand annealing (SDSA) mechanisms [176]. However, only an HJ pathway results in crossing-over [176]. At the pachytene stage, chromatin decondensation in hot spots is promoted. A loosening of DNA structure allows the enzymes to bind properly to DSB sites. Acetylation of H3K9, H3K14, and H3K56 is responsible for relaxation and recruitment of repair enzymes [189]. Additionally, ubiquitination H2AK119 intensifies, and H2AK13-15 start to emerge. Those alterations take part in the recruitment of repair proteins, such as RAP80 (receptor-associated protein 80), BRCA1 (breast cancer type 1 susceptibility protein), and 53BP1 (tumor protein p53 binding protein 1) [190]. Finished DSB repair marks the end of homologous recombination (HR) in meiosis [176].

On the other hand, MSCI starts to emerge in the pachytene [177]. MSCI is a meiotic silencing mechanism of asynapsed chromatin (MSUC) that affects only X and Y chromosomes. MSUC action ensures that asynapsed regions of autosomes are not transcriptionally active [177]. MSCI performs the same function, but it occurs in the sex chromosomes, and it is responsible for creating the sex body, also known as XY body [177]. In pachytene, chromatin condensation and gene repression are promoted by the reduction of histone modifications responsible for marking histones with activation epimarks on sex chromosomes [188]. Among those, the most prominent is a reduction of acetylation at: H3K9, H4K12, and H4K16, and reduction of methylation at H3K4me3 [188]. In contrast, repressive modifications like: methylation at H3K9 and H3K4, and hyperacetylation at H3K5, and H3K8 are present [188]. In pachytene, the third wave of γH2AX phosphorylation occurs on asynapsed fragments of sex chromosomes [76, 177]. DNA damage response precedes yH2AX phosphorylation [191]. First, BRCA1 accumulates on asynapsed sex chromosome axes and allows the recruitment of TOPBP1 (DNA topoisomerase 2-binding protein 1) and ATR kinase. In the next step, the yH2AX phosphorylation by ATR occurs, which in consequence attracts MDC1 (mediator of DNA damage checkpoint protein 1) [191]. MDC1 then coordinates spread of yH2AX to the subsequent nucleosomes [191]. The absence of γH2AX modification on sex chromosomes leads to defects in XY synapsis, MSCI failure, and complete arrest in the pachytene stage [177, 191]. Additionally, to ensure that gene silencing will persist, new histone variants are introduced from pachytene onwards. One of them is H2A.Z, which replaces canonical H2A histone [126].

The so-called: ‘pachytene checkpoint’ occurs in the late pachytene stage [192]. In response to HR or synapsis formation failures, the pachytene checkpoint is involved in arrest or delaying of spermatocytes progress through the pachytene stage. This mechanism prevents the formation of aneuploid spermatids [192]. Dot1 histone methyltransferase is responsible for the methylation of lysine 79 residue in histone H3 (H3K79me), which controls this checkpoint. The absence of Dot enzyme decreases the level of H3K79me, and thus leads to meiotic arrest at the pachytene stage of prophase I [192, 193]. This quality-control mechanism is well characterized in yeast and is evolutionary-conserved from yeast to mammals [192].

The activation of some genes located on sex chromosomes (~ 13%) is necessary for the later phases of meiosis and spermiogenesis [194, 195]; however, the MSCI process inactivates the expression of all genes located within the sex body [194, 196]. In the dividing cell, special mechanisms allow certain genes to be re-expressed, and this reactivation starts at the pachytene stage. Two major changes in the histone code are responsible for this reactivation. The first of them is the replacement of the H2A histone with H2A.B.3 (known also as H2A.Lap1) variant, which is mouse orthologue of human H2A.B variant [194, 197]. This reinstatement occurs around transcription start sites (TSS), and is associated with the relaxation of chromatin structure (less compacted nucleosome states) and gene transcription [194, 197, 198]. This histone variant is selectively located within X chromosome genes active in round spermatids [194]. The second modification that helps to restore the transcriptional activity later in spermatogenesis, is the polyubiquitination of an unknown substrate by RNF8 (ring finger protein 8) with deubiquitination of H2AK119 by SCML2 (Scm polycomb group protein like 2) [97]. As shown in mouse study, those modifications promote H3K27 acetylation of the enhancer region of escape genes in the late pachytene stage, and H3K4me2 of the promoter region in early diplotene. Histone ubiquitination is removed from sex chromosomes at the late diplotene stage, while both H3K27ac and H3K4me2 persist in spermiogenesis as an epigenetic memory, where they facilitate the activation of escape genes such as *Gm9* (predicted gene 9) or *Prdx4* (peroxiredoxin 4) [97].

### Diplotene and diakinesis

From diplotene through metaphase, bivalent methylation/phosphorylation post-translational modifications will be imposed, namely: H3K9me2/H3S10ph and H3K27me2/H3S28ph [199]. Those modifications could facilitate specific condensed chromatin conformation during meiosis, as well as mitosis [199]. Additionally, the presence of H3K9me2/3 on sex chromosomes ensures chromatin condensation of XY chromosomes, which is necessary for MSCI persistence and transition to postmeiotic status of sex chromosomes [200]. Starting from diplotene until the end of meiosis, histones H3 and H4 are underacetylated on sex chromosomes, except for H4K16 throughout the X and Y chromosomes, and H4K5 only in the pericentromeric region [188].

After the end of prophase I, primary spermatocytes complete the following phases of the first meiotic division: metaphase I, anaphase I, telophase I and cytokinesis, and become secondary spermatocytes. Secondary spermatocytes quickly finish the second meiotic division, which results in haploid spermatids.

## Spermiogenesis

In a process of spermiogenesis, the last step is the transformation of round spermatids into sperm cells, which are the final differentiation point of male reproductive cells [201]. During this transformation, immature spermatids undergo chromatin condensation, acrosome and tail formation, elongation, and cytoplasm reduction [201]. This transformation is possible due to histone-to-protamine exchange, which is the most important event performed during spermiogenesis. Transition nuclear proteins (TNPs: TNP1 and TNP2) mediate the transition from histones to protamines. In the first step, TNPs substitute histones, and are later replaced by protamines [134, 201, 202]. Protamines are small, arginine-rich proteins, expressed specifically in spermiogenesis starting from elongating spermatids onwards [203]. In mammals, two protamines P1 and P2 (in human encoded by *PRM* and *PRM2*, respectively) are incorporated into the chromatin structure. The arginine-rich DNA-anchoring domain allows protamines to wrap around the major groove of DNA helix. This neutralizes the negative charge of DNA backbone and allows nucleo-protamine chromatin to be coiled and condensed much tighter than nucleo-histone chromatin type of packaging [203]. Most of the sperm chromatin is associated with protamines [204]. In mouse sperm cells, only 1% of the whole genome remains attached to histones, while in humans, the histones are present in 10%-15% of the sperm chromatin [35, 134, 204, 205]. Disturbances in ratio between particular protamines or between protamines and histones lead to decrease of semen parameters and to reproductive problems [134, 205, 206]. The remaining nucleo-histone chromatin is present principally around the centromeric and telomeric regions of the chromosomes [204, 207]. The main histone variants and their involvement in modifications during the histone-to-protamine exchange, followed by relevant mouse models have been summarized in [134].

Histone acetylation is essential for the histone-to-protamine transition. H4K5, H4K8, and H4K12 acetylation are the first markers of the histone H4 hyperacetylation, which facilitates chromatin opening at the early stages of spermatid elongation [90, 208, 209]. In mouse round spermatids, the histone H4 phosphorylation of the serine 1 residue (H4S1) forego hyperacetylation [210]. This modification may help to compact DNA before histone-to-protamine transition [211]. Additionally, histone H3 hyperacetylation, mainly at lysine 9, 18, 23 residues (H3K9ac, H3K18ac, H3K23ac), has been demonstrated in spermatids, but its role in histone-to-protamine exchange is less prominent [166].

Thanks to histone hyperacetylation, DNA topoisomerase II beta (TOP2β) binds to DNA and induces DSB formation [212]. Similar to HR and MSCI, both ATM and ATR enzymes catalyze the formation of histone yH2AX, but during spermiogenesis also additional protein kinase TSSK6 (testis-specific serine/threonine kinase) is required [12]. yH2AX then binds to MDC1 protein, which facilitates ubiquitination of histones H2A and H2B by recruitment of RNF8 E3 ubiquitin ligase [96, 117]. Additionally, MDC1 replaces H2A/B histones for a H2AZ/H2B dimer [117]. Those modifications lead to chromatin opening and promote H4K16ac by MOF histone acetyltransferase [96, 213]. H4K16 acetylation indicates global histone removal [96]. This process is performed by bromodomain testis-specific protein (BRDT), which binds to tetra-acetylated H4 histone and guides the histone removal [208, 214, 215].

In elongating spermatids, histone methylation is also present, and mainly consists of H3K4me2/3, H3K9me2/3, H3K27me3, and H3K79me2/3 [117, 193]. During this stage, both repressive and activating histone modifications are present. Activating H3K4me2/3 helps in the ubiquitination of histone H2A by providing a binding place for PHD finger protein 7 (PHF7) E3 ubiquitin ligase [213]. In round spermatids, H3K4me1/2/3 is located in euchromatin and activates gene expression of genes important for spermiogenesis [117]. Then in elongating spermatids, H3K9me1/2 and H3K36me3 regulate the expression of genes coding transition nuclear proteins and protamines. H3K9me1/2 presence in the promoter region of those genes downregulates their expression [93], while H3K36me3 acts in the opposite manner [98].

After final changes in chromatin compaction and necessary epigenetic modifications, the non-motile elongated spermatids are released into the lumen of the seminiferous tubules in the process of spermiation. Next, during the transition through epididymis, the contact with unique microenvironmental lumen factors leads to the maturation of the sperm cells – assessment of motility and ability to fertilize [216–220].

## Conclusions

The ever-growing number of studies of epigenetic changes in both male and female gametogenesis leads to the detailed understanding of the mechanisms governing the contribution and teamwork of the paternal and maternal genomes during the fertilization process and embryo development, also at the level of genetic and epigenetic cooperation. In this review, we have summarized the most important epigenetic modifications of DNA and histones crucial for the process of spermatogenesis. Following the facts, that each step of spermatogenesis is characterized by determined epimarks, and the complexity of male gametogenesis is intricated, the important role of epigenetic changes in male germ cell development is clearly highlighted. It is also significant in the context of male reproductive failures, with unknown or unclear genetic background, so far, and the rising number of cases with epigenetic background of the infertility as the main reason.

It is known that the majority of data have been provided from mouse studies. It is caused by the fact that still there is a low availability of human material from particular gametogenesis stages (incl. ethical issues) or embryonal ones. There is also no availability (or are problems with culturing) for cell lines related to human spermatogenesis that could be used for genetic or epigenetic research. Of course, even if it is known that mice phenotype of predicted model is often with a less severe phenotype that human (because of alternative ways of compensation for the loss of a proper protein), methylation patterns and reprogramming events are relatively conserved between human and mouse and mouse still is a good model organism for inferring general mechanisms. Translation of mouse model results into human male infertility enhances understanding of fertility pathways, and is able to mimick some aspects of primary human infertility (examples of the mouse-to-human modelling has been widely reviewed in [221, 222]). It seems to be important to expand further studies and to involve mouse models also for epididymis evaluation in the epigenetic manner. The increasing number of data indicates changes of epigenetic markers of sperm DNA also during the maturation in epididymis (and its particular parts), and thus puts attention also on the epigenetic role of this organ, leading to the need of revision of the statement that the histone PTMs are completed before the release of the sperm from the male gonad [21, 217, 223]. So, in the context of male reproduction there is a strong need to deep evaluation of the epididymal processes in the epigenetic manner, particularly in the light of the known rich epididymal microenvironment and its already described influence both: on sperm maturation (motility, fertilization capability), as well as on the rate of sperm DNA damage [21, 202, 217–220, 223]. Thus, the link between proper epimarks and sperm chromatin integrity perhaps will possess part of its principles also from the epididymal side. Additionally, the adult diseases of the offspring linked to paternal transmission of dysfunctions related to epigenetic patterns (i.e., obesity), should also be evaluated more extensively, with the special attention to the environmental influence and the life style of the future father [68, 224–226].

From the clinical view point, the sperm epimarks should also be checked for azoospermic or cryptozoospermic patients subjected to fertilization with gametes aspirated from testicular biopsy – one step before epididymal maturation. Maybe there would be an answer for cases with successful fertilization rate (= we have an embryo) but unsuccessful embryo development (why it is not developing, when preimplantation genetics is fine), aberrant imprinting patterns, birth defects or poor health outcome of ART-born children [222, 224–227]. Thus, there is a strong need for further evaluation of epigenetic marks and mechanisms in male reproductive context, not only on human samples but also with the mouse models, because of the fact that animal studies give better accessibility of biological samples to develop a great variety of experimental pathways (specifically in testis-derived gametes and in embryo evaluation after ART). Recently expanding range of high-resolution methods and big data analyses seem to give great capabilities of a detailed and complex data acquirement at the single cell level, including genetic and epigenetic data among the whole genome and methylome, and thus leading to getting of priceless evidences for reproduction, and also for developing of novel routes for disease aetiology and its prevention or treatment in the future. The exploration of this area concerning potential linkage between male reproductive epigenome and infertility or other disease phenotypes in the offspring (not necessarily related to fertility) should be more extensive in a practical and theoretical challenges.

## Data Availability

Authors can confirm that all relevant data are included in the article.

## Abbreviations

1n: Haploid genome
2n: Diploid genome
4n: Tetraploid genome
53BP1: Tumor protein p53 binding protein 1
5caC: 5-Carboxy-cytosine
5fC: 5-Formyl-cytosine
5hmC: 5-Hydroxymethyl-cytosine
5mC: 5-Methyl-cytosine
ac: Acetylation
ART: Artificial reproductive technique
ATP: Adenosine triphosphate
BER: Base excision repair
BRCA1: Breast cancer type 1 susceptibility protein
BRDT: Bromodomain testis associated
BRDT: Bromodomain testis-specific protein
Camk4: Calcium/calmodulin-dependent protein kinase IV
Cdyl: Chromodomain protein, Y chromosome-like
Cfp1: CXXC finger 1
Chd5: Chromodomain helicase DNA binding protein 5
Dazl: Deleted in azoospermia-like
Ddx4: DEAD-box helicase 4
Dim5: Defective in methylation-5
DLK1-DIO3: Delta like non-canonical Notch ligand 1 – iodothyronine deiodinase 3
DMRs: Differentially methylated regions
DNA: Deoxyribonucleic acid
Dnmt3a: DNA methyltransferase 3A
Dnmt3l: DNA (cytosine-5-)-methyltransferase 3-like
DNMTs: DNA methyltransferases
DOT1L: Disruptor of telomeric silencing-1-like
Dot1l: DOT1-like, histone H3 methyltransferase
DSBs: Double-strand breaks
DUBs: Deubiquitinating enzymes
E1: Ubiquitin-activating enzyme
E2: Ubiquitin-conjugating enzyme
E3: Ubiquitin ligase
Epc1: Enhancer of polycomb homolog 1
Fancd2: Fanconi anemia, complementation group D2
Fanci: Fanconi anemia, complementation group I
Fbxl10: Lysine (K)-specific demethylase 2B
G9A: Euchromatic histone-lysine N-methyltransferase 2 gene
Gfap: Glial fibrillary acidic protein gene
Gm9: Predicted gene 9
H19-IGF2: H19 imprinted maternally expressed transcript – insulin like growth factor 2
HATs: Histone acetyltransferases
Hdac3: Histone deacetylase 3
Hdac6: Histone deacetylase 6
HDACs: Histone deacetylases
HJs: Holliday junctions
HKMTs: Lysine methyltransferases
HMTs: Histone methyltransferases
Hox: Homeobox proteins gene
HR: Homologous recombination
Hr6b: Ubiquitin-conjugating enzyme E2B
Jhmd2a/ Jmjd1a: Lysine (K)-specific demethylase 3A (Kdm3a)
JmJd1c: Jumonji domain containing 1C
Kdm1a: Lysine (K)-specific demethylase 1A
Kdm4d: Lysine (K)-specific demethylase 4D
LEFTY: Left–right determination factor
lncRNA: Long non-coding RNA
MDC1: Mediator of DNA damage checkpoint protein 1
me: Methylation
Mettl21a: Methyltransferase like 21A
miRNA: MicroRNA
Mll2: Lysine (K)-specific methyltransferase 2D
KAT8: Lysine acetyltransferase 8
MOF: Male absent on the first
MRN: Mre11, Rad50, and Nbs1
MSCI: Meiotic sex chromosome inactivation
MSUC: Meiotic silencing mechanism of asynapsed chromatin
Mthfr: Methylenetetrahydrofolate reductase
Mtrr: 5-methyltetrahydrofolate-homocysteine methyltransferase reductase
NANOG: Nanog homeobox
ncRNA: Non-coding RNA
Nsd1: Nuclear receptor-binding SET-domain protein 1
OCT4: Octamer-binding transcription factor 4
PADI4: Protein-arginine deiminase type-4
Parp1/2: Poly (ADP-ribose) polymerase family, member 1/2
PGCs: Primordial germ cells
ph: Phosphorylation
PHF7: PHD finger protein 7
piRNA: Piwi-interacting RNA
PLZF: Promyelocytic leukaemia zinc finger transcriptional repressor
Prdm9: PR domain containing 9
PRDM9: PR/SET domain 9
Prdx4: Peroxiredoxin 4
PRM1: Protamine 1
PRM2: Protamine 2
PRMD14: PR/SET domain 14
Prmt1: Protein arginine N-methyltransferase 1
Prmt5: Protein arginine methyltransferase 5
Prmt5: Protein arginine N-methyltransferase 5
Prmt7: Protein arginine N-methyltransferase 7
PRMTs: Protein arginine methyltransferases
Ptip: PAX interacting (with transcription-activation domain) protein 1
PTMs: Post-transcriptional modifications
Pygo2: Pygopus 2
RAP80: Receptor-associated protein 80
RNA: Ribonucleic acid
RNF20: Ring finger protein 20
RNF8: Ring finger protein 8
rRNA: Ribosomal RNA
SAM: S-adenosyl-L-methionine
SCML2: Scm polycomb group protein like 2
SDSA: Synthesis-dependent strand annealing
SET: Su(var)3–9, Enhancer-of-zeste and Trithorax
Setd2: SET domain containing 2
Setdb1: SET domain, bifurcated 1
siRNA: Small interfering RNA
SirT1: Sirtuin 1
Sly: Sycp3 like Y-linked
snoRNA: Small nucleolar RNA
snRNA: Small nuclear RNA
SOX2: SRY-box transcription factor 2
SPC: Spermatocyte
SPD: Spermatid
SPG A: Spermatogonia A
SPG Ad: Spermatogonia A dark
SPG Ap: Spermatogonia A pale
SPG B: Spermatogonia B
Spo11: SPO11 initiator of meiotic double stranded breaks
SSCs: Spermatogonial stem cells
STELLA: Developmental pluripotency-associated protein 3 gene
Stra8: Stimulated by retinoic acid gene 8
SUMO: Small ubiquitin-like modifiers
Suv39h1/2: Suppressor of variegation 3-9 1/2
SYCP3: Synaptonemal complex protein 3
TDG: Thymine-DNA glycosylase
TET: Ten-Eleven Translocation
Tet1: Tet methylcytosine dioxygenase 1
TEX19: Testis expressed 19
Tip60: Lysine acetyltransferase 5 (Kat5)
TNPs: Transition nuclear proteins
TOP2β: DNA topoisomerase II beta
TOPBP1: DNA topoisomerase 2-binding protein 1
tRNA: Transfer RNA
Tssk6: Testis-specific serine kinase 6
TSSs: Transcription start sites
ub: Ubiquitination
Ubr2: Ubiquitin protein ligase E3 component n-recognin 2
UHRF1: Ubiquitin-like with PHD and ring finger domains 1 gene
Utx: Lysine (K)-specific demethylase 6A

## References

[1] Handy DE, Castro R, Loscalzo J (2011). Epigenetic modifications: basic mechanisms and role in cardiovascular disease. Circulation.

[2] Meyer RG, Ketchum CC, Meyer-Ficca ML (2017). Heritable sperm chromatin epigenetics: A break to remember. Biol Reprod.

[3] Ben Maamar M, Sadler-Riegelman Beck D (2018). Skinner MK Epigenetic transgenerational inheritance of altered sperm histone retention sites. Sci Rep.

[4] Xavier MJ, Roman SD, Aitken RJ, Nixon B (2019). Transgenerational inheritance: How impacts to the epigenetic and genetic information of parents affect offspring health. Hum Reprod Update.

[5] Allis CD, Jenuwein T (2016). The molecular hallmarks of epigenetic control. Nat Rev Genet.

[6] Peixoto P, Cartron P-F, Serandour AA, Hervouet E (2020). From 1957 to Nowadays: A Brief History of Epigenetics. Int J Mol Sci.

[7] Luo C, Hajkova P, Ecker JR (2018). Dynamic DNA methylation: In the right place at the right time. Science.

[8] Moore LD, Le T, Fan G (2013). DNA methylation and its basic function. Neuropsychopharmacology.

[9] Jang HS, Shin WJ, Lee JE, Do JT (2017). CpG and Non-CpG Methylation in Epigenetic Gene Regulation and Brain Function. Genes (Basel).

[10] Zhang Y, Reinberg D (2001). Transcription regulation by histone methylation: interplay between different covalent modifications of the core histone tails. Genes Dev.

[11] Patel DJ, Wang Z (2013). Readout of epigenetic modifications. Annu Rev Biochem.

[12] Jha KN, Tripurani SK, Johnson GR (2017). TSSK6 is required for γH2AX formation and the histone-to-protamine transition during spermiogenesis. J Cell Sci.

[13] Sabari BR, Zhang D, Allis CD, Zhao Y (2017). Metabolic regulation of gene expression through histone acylations. Nat Rev Mol Cell Biol.

[14] Wei J-W, Huang K, Yang C, Kang C-S (2017). Non-coding RNAs as regulators in epigenetics (Review). Oncol Rep.

[15] Morey C, Avner P (2004). Employment opportunities for non-coding RNAs. FEBS Lett.

[16] Stewart KR, Veselovska L, Kelsey G (2016). Establishment and functions of DNA methylation in the germline. Epigenomics.

[17] Allegrucci C, Thurston A, Lucas E, Young L (2005). Epigenetics and the germline. Reproduction.

[18] Hammoud SS, Nix DA, Zhang H, Purwar J, Carrell DT, Cairns BR (2009). Distinctive chromatin in human sperm packages genes for embryo development. Nature.

[19] Ge S-Q, Lin SL, Zhao ZH, Sun QY (2017). Epigenetic dynamics and interplay during spermatogenesis and embryogenesis: Implications for male fertility and offspring health. Oncotarget.

[20] Carrell DT (2012). Epigenetics of the male gamete. Fertil Steril.

[21] Castillo J, Jodar M, Oliva R (2018). The contribution of human sperm proteins to the development and epigenome of the preimplantation embryo. Hum Reprod Update.

[22] Menezo YJR, Silvestris E, Dale B, Elder K (2016). Oxidative stress and alterations in DNA methylation: Two sides of the same coin in reproduction. Reprod BioMed Online.

[23] Benchaib M, Braun V, Ressnikof D, Lornage J, Durand P, Niveleau A (2005). Influence of global sperm DNA methylation on IVF results. Hum Reprod.

[24] Feinberg JI, Bakulski KM, Jaffe AE, Tryggvadottir R, Brown SC, Goldman LR (2015). Paternal sperm DNA methylation associated with early signs of autism risk in an autism-enriched cohort. Int J Epidemiol.

[25] Stuppia L, Franzago M, Ballerini P, Gatta V, Antonucci I (2015). Epigenetics and male reproduction: The consequences of paternal lifestyle on fertility, embryo development, and children lifetime health. Clin Epigenet.

[26] White CR, Denomme MM, Tekpetey FR, Feyles V, Power SGA, Mann MRW (2015). High frequency of imprinted methylation errors in human preimplantation embryos. Sci Rep.

[27] Miller D, Brinkworth M, Iles D (2010). Paternal DNA packaging in spermatozoa: More than the sum of its parts? DNA, histones, protamines and epigenetics. Reproduction.

[28] Aberg KA, McClay JL, Nerella S, Clark S, Kumar G, Chen W (2014). Methylome-wide association study of schizophrenia: Identifying blood biomarker signatures of environmental insults. JAMA Psychiat.

[29] Bennett DA, Yu L, Yang J, Srivastava GP, Aubin C, De Jager PL (2015). Epigenomics of Alzheimer’s disease. Transl Res.

[30] Wei Y, Schatten H, Sun Q-Y (2015). Environmental epigenetic inheritance through gametes and implications for human reproduction. Hum Reprod Update.

[31] Ioannou D, Miller D, Griffin DK, Tempest H (2016). Impact of sperm DNA chromatin in the clinic. J Assist Reprod Genet.

[32] El Hajj N, Haaf T (2013). Epigenetic disturbances in in vitro cultured gametes and embryos: Implications for human assisted reproduction. Fertil Steril.

[33] Camprubi C, Salas-Huetos A, Aiese-Cigliano R, Godo A, Pons M-C, Castellano G (2016). Spermatozoa from infertile patients exhibit differences of DNA methylation associated with spermatogenesis-related processes: An array-based analysis. Reprod Biomed Online.

[34] Wyns C, Bergh C, Calhaz-Jorge C, De Geyter C, Kupka MS, Motrenko T (2020). The European IVF–monitoring Consortium (EIM) for the European Society of Human Reproduction and Embryology (ESHRE) (2020) ART in Europe, 2016: Results generated from European registries by ESHRE. Hum Reprod Open..

[35] Olszewska M, Kordyl O, Kamieniczna M, Fraczek M, Jedrzejczak P, Kurpisz M (2022). Global 5mC and 5hmC DNA levels in human sperm subpopulations with differentially protaminated chromatin in normo- and oligoasthenozoospermic males. Int J Mol Sci.

[36] Rotondo JC, Lanzillotti C, Mazziotta C, Tognon M, Martini F (2021). Epigenetics of male infertility: the role of DNA methylation. Front Cell Dev Biol.

[37] Lambrot R, Chan D, Shao X, Aarabi M, Kwan T, Bourque G (2021). Whole-genome sequencing of H3K4me3 and DNA methylation in human sperm reveals regions of overlap linked to fertility and development. Cell Rep.

[38] Lambrot R, Siklenka K, Lafleur C, Kimmins S (2019). The genomic distribution of histone H3K4me2 in spermatogonia is highly conserved in sperm. Biol Reprod.

[39] Olszewska M, Barciszewska MZ, Fraczek M, Huleyuk N, Chernykh VB, Zastavna D (2017). Global methylation status of sperm DNA in carriers of chromosome structural aberrations. Asian J Androl.

[40] Benchaib M, Ajina M, Lornage J, Niveleau A, Durand P, Guerin JF (2003). Quantitation by image analysis of global DNA methylation in human spermatozoa and its prognostic value in in vitro fertilization: A preliminary study. Fertil Steril.

[41] Jenkins TG, Aston KI, Hotaling JM, Shamsi MB, Simon L, Carrell DT (2016). Teratozoospermia and asthenozoospermia are associated with specific epigenetic signatures. Andrology.

[42] Jenkins TG, Aston KI, Meyer TD, Hotaling JM, Shamsi MB, Johnstone EB (2016). Decreased fecundity and sperm DNA methylation patterns. Fertil Steril..

[43] Jenkins TG, Aston KI, Cairns B, Smith A, Carrell DT (2018). Paternal germ line aging: DNA methylation age prediction from human sperm. BMC Genom.

[44] Kobayashi H, Hiura H, John RM, Sato A, Otsu E, Kobayashi N (2009). DNA methylation errors at imprinted loci after assisted conception originate in the parental sperm. Eur J Hum Genet.

[45] Krausz C, Riera-Escamilla A (2018). Genetics of male infertility. Nat Rev Urol..

[46] Sujit KM, Sarkar S, Singh V, Pandey R, Agrawal NK, Trivedi S (2018). Genome-wide differential methylation analyses identifies methylation signatures of male infertility. Hum Reprod.

[47] Luján S, Caroppo E, Niederberger C, Arce JC, Sadler-Riggleman I, Beck D (2019). Sperm DNA Methylation Epimutation Biomarkers for Male Infertility and FSH Therapeutic Responsiveness. Sci Rep.

[48] Neto FTL, Bach PV, Najari BB, Li PS, Goldstein M (2016). Spermatogenesis in humans and its affecting factors. Semin Cell Dev Biol.

[49] Hess RA, Renato de Franca L (2008). Spermatogenesis and cycle of the seminiferous epithelium. Adv Exp Med Biol..

[50] Griswold MD (2016). Spermatogenesis: The Commitment to Meiosis. Physiol Rev.

[51] Clermont Y (1972). Kinetics of spermatogenesis in mammals: seminiferous epithelium cycle and spermatogonial renewal. Physiol Rev.

[52] Amann RP (2008). The cycle of the seminiferous epithelium in humans: a need to revisit?. J Androl.

[53] Heller CH, Clermont Y (1964). Kinetics of the germinal epithelium in man. Recent Prog Horm Res.

[54] Toshimori K (2003). Biology of spermatozoa maturation: an overview with an introduction to this issue. Microsc Res Tech.

[55] NCBI – National Center for Biotechnology Information: https://www.ncbi.nlm.nih.gov/gene/

[56] MGI – Mouse Genome Informatics: http://www.informatics.jax.org

[57] Mansour AA, Gafni O, Weinberger L, Zviran A, Ayyash M, Rais Y (2012). The H3K27 demethylase Utx regulates somatic and germ cell epigenetic reprogramming. Nature.

[58] Kelly TLJ, Neaga OR, Schwahn BC, Rozen R, Trasler JM (2005). Infertility in 5,10-Methylenetetrahydrofolate Reductase (MTHFR)-Deficient Male Mice Is Partially Alleviated by Lifetime Dietary Betaine Supplementation. Biol Reprod.

[59] Chan D, Cushnie DW, Neaga OR, Lawrance AK, Rozen R, Trasler JM (2010). Strain-Specific Defects in Testicular Development and Sperm Epigenetic Patterns in 5,10-Methylenetetrahydrofolate Reductase-Deficient Mice. Endocrinology.

[60] Shirane K, Miura F, Ito T, Lorincz MC (2020). NSD1-deposited H3K36me2 directs de novo methylation in the mouse male germline and counteracts Polycomb-associated silencing. Nat Genet.

[61] Chen M, Wang Y, Lin L, Dong F, Wu H, Bao S (2020). PRMT7 is involved in regulation of germ cell proliferation during embryonic stage. Biochem Biophys Res Communications.

[62] Kim S, Günesdogan U, Zylicz J, Hackett JA, Cougot D, Bao S (2014). PRMT5 Protects Genomic Integrity during Global DNA Demethylation in Primordial Germ Cells and Preimplantation Embryos. Mol Cell.

[63] Liu S, Karimi MM, Shirane K, Bogutz A, Lefebvre L, Brind’Amour J (2014). Setdb1 is required for germline development and silencing of H3K9me3-marked endogenous retroviruses in primordial germ cells. Genes Dev..

[64] Kaneda M, Okano M, Hata K, Sado T, Tsujimoto N, Li E (2004). Essential role for de novo DNA methyltransferase Dnmt3a in paternal and maternal imprinting. Nature.

[65] Dura M, Teissandier A, Armand M, Barau J, Lapoujade C, Fouchet P (2022). DNMT3A-dependent DNA methylation is required for spermatogonial stem cells to commit to spermatogenesis. Nat Genet.

[66] Xu L, Xu W, Li D, Yu X, Gao F, Qin Y (2021). FANCI plays an essential role in spermatogenesis and regulates meiotic histone methylation. Cell Death Dis.

[67] Myrick DA, Christopher MA, Scott AM, Simon AK, Donlin-Asp PG, Kelly WG (2017). KDM1A/LSD1 regulates the differentiation and maintenance of spermatogonia in mice. PLoS ONE.

[68] Lismer A, Siklenka K, Lafleur C, Dumeaux V, Kimmins S (2020). Sperm histone H3 lysine 4 trimethylation is altered in a genetic mouse model of transgenerational epigenetic inheritance. Nucleic Acid Res.

[69] Glaser S, Lubitz S, Loveland KL, Ohbo K, Robb L, Schwenk F (2009). The histone 3 lysine 4 methyltransferase, Mll2, is only required briefly in development and spermatogenesis. Epigenet Chromatin.

[70] Huang G, Liu L, Wang H, Gou M, Gong P, Tian C (2020). Tet1 Deficiency Leads to Premature Reproductive Aging by Reducing Spermatogonia Stem Cells and Germ Cell Differentiation. iScience..

[71] Ki BS, Shim SH, Park C, Yoo H, La H, Lee O-H (2022). Epigenetic regulator Cfp1 safeguards male meiotic progression by regulating meiotic gene expression. Exp Mol Med.

[72] Webster KE, O'Bryan MK, Fletcher S, Crewther PE, Aapola U, Craig J (2005). Meiotic and epigenetic defects in Dnmt3L-knockout mouse spermatogenesis. Proc Nat Acad Sci.

[73] Tachibana M, Nozaki M, Takeda N, Shinkai Y (2007). Functional dynamics of H3K9 methylation during meiotic prophase progression. EMBO J.

[74] Yin H, Kang Z, Zhang Y, Gong Y, Liu M, Xue Y (2021). HDAC3 controls male fertility through enzyme-independent transcriptional regulation at the meiotic exit of spermatogenesis. Nuc Acids Res.

[75] Baarends WM, Wassenaar E, Hoogerbrugge JW, van Cappellen G, Roest HP, Vreeburg J (2003). Loss of HR6B Ubiquitin-Conjugating Activity Results in Damaged Synaptonemal Complex Structure and Increased Crossing-Over Frequency during the Male Meiotic Prophase. Mol Cell Biol.

[76] Jiang H, Gao Q, Zheng W, Yin S, Wang L, Zhong L (2018). MOF influences meiotic expansion of H2AX phosphorylation and spermatogenesis in mice. PLoS Genet.

[77] Ozawa M, Fukuda T, Sakamoto R, Honda H, Yoshida N (2016). The Histone Demethylase FBXL10 Regulates the Proliferation of Spermatogonia and Ensures Long-Term Sustainable Spermatogenesis in Mice. Biol Reprod.

[78] Liu C. Epigenetic Role of PTIP in Mouse Spermatogenesis. A thesis submitted to the Graduate College of Marshall University. 2015. https://mds.marshall.edu/cgi/viewcontent.cgi?article=1928&context=etd

[79] Diagouraga B, Clément JAJ, Duret L, Kadlec J, de Massy B, Baudat F (2018). PRDM9 Methyltransferase Activity Is Essential for Meiotic DNA Double-Strand Break Formation at Its Binding Sites. Mol Cell.

[80] Waseem S, Kumar S, Lee K, Yoon B-H, Kim M, Kim H (2021). Protein Arginine Methyltransferase 1 Is Essential for the Meiosis of Male Germ Cells. Int J Mol Sci.

[81] Xu Z, Song Z, Li G, Tu H, Liu W, Liu Y, et al. H2B ubiquitination regulates meiotic recombination by promoting chromatin relaxation. Nuc Acids Res. 2016;44(20):9681-97. 10.1093/nar/gkw652 PMC5175339 27431324

[82] Cheng E-C, Hsieh C-L, Liu N, Wang J, Zhong M, Chen T (2021). The Essential Function of SETDB1 in Homologous Chromosome Pairing and Synapsis during Meiosis. Cell Rep.

[83] Hirota T, Blakeley P, Sangrithi MN, Mahadevaiah SK, Encheva V, Snijders AP (2018). SETDB1 Links the Meiotic DNA Damage Response to Sex Chromosome Silencing in Mice. Dev Cell.

[84] Peters AHFM, O'Carroll D, Scherthan H, Mechtler K, Sauer S, Schöfer C (2001). Loss of the Suv39h Histone Methyltransferases Impairs Mammalian Heterochromatin and Genome Stability. Cell.

[85] An JY, Kim E-A, Jiang Y, Zakrzewska A, Kim DE, Lee MJ (2010). UBR2 mediates transcriptional silencing during spermatogenesis via histone ubiquitination. Proc Nat Acad Sci.

[86] Dong J, Wang X, Cao C, Wen Y, Sakashita A, Chen S (2019). UHRF1 suppresses retrotransposons and cooperates with PRMT5 and PIWI proteins in male germ cells. Nat Commun.

[87] Wu JY, Ribar TJ, Cummings DE, Burton KA, McKnight GS, Means AR (2000). Spermiogenesis and exchange of basic nuclear proteins are impaired in male germ cells lacking Camk4. Nat Genet.

[88] Liu S, Yu H, Liu Y, Liu X, Zhang Y, Bu C (2017). Chromodomain Protein CDYL Acts as a Crotonyl-CoA Hydratase to Regulate Histone Crotonylation and Spermatogenesis. Mol Cell.

[89] Zhuang T, Hess RA, Kolla V, Higashi M, Raabe TD, Brodeur GM (2014). CHD5 is required for spermiogenesis and chromatin condensation. Mech Dev.

[90] Dong Y, Isono K-i, Ohbo K, Endo TA, Ohara O, Maekawa M (2017). EPC1/TIP60-Mediated Histone Acetylation Facilitates Spermiogenesis in Mice. Mol Cell Biol..

[91] Nakajima R, Okano H, Noce T (2016). JMJD1C Exhibits Multiple Functions in Epigenetic Regulation during Spermatogenesis. PLoS ONE.

[92] Okada Y, Scott G, Ray MK, Mishina Y, Zhang Y (2007). Histone demethylase JHDM2A is critical for Tnp1 and Prm1 transcription and spermatogenesis. Nature.

[93] Liu Z, Zhou S, Liao L, Chen X, Meistrich M, Xu J (2010). Jmjd1a demethylase-regulated histone modification is essential for cAMP-response element modulator-regulated gene expression and spermatogenesis. J Biol Chem.

[94] Meyer-Ficca ML, Ihara M, Lonchar JD, Meistrich ML, Austin CA, Min W (2011). Poly(ADP-ribose) Metabolism Is Essential for Proper Nucleoprotein Exchange During Mouse Spermiogenesis1. Biol Reprod.

[95] Nair M, Nagamori I, Sun P, Mishra DP, Rhéaume C, Li B (2008). Nuclear regulator Pygo2 controls spermiogenesis and histone H3 acetylation. Dev Biol.

[96] Lu L-Y, Wu J, Ye L, Gavrilina GB, Saunders TL, Yu X (2010). RNF8-dependent histone modifications regulate nucleosome removal during spermatogenesis. Dev Cell.

[97] Adams SR, Maezawa S, Alavattam KG, Abe H, Sakashita A, Shroder M (2018). RNF8 and SCML2 cooperate to regulate ubiquitination and H3K27 acetylation for escape gene activation on the sex chromosomes. PLoS Genet.

[98] Zuo X, Rong B, Li L, Lv R, Lan F, Tong M-H (2018). The histone methyltransferase SETD2 is required for expression of acrosin-binding protein 1 and protamines and essential for spermiogenesis in mice. J Biol Chem.

[99] Bell EL, Nagamori I, Williams EO, Del Rosario AM, Bryson BD, Watson N (2014). SirT1 is required in the male germ cell for differentiation and fecundity in mice. Development.

[100] Moretti C, Serrentino M-E, Ialy-Radio C, Delessard M, Soboleva TA, Tores F (2017). SLY regulates genes involved in chromatin remodeling and interacts with TBL1XR1 during sperm differentiation. Cell Death Differ.

[101] Uysal F, Akkoyunlu G, Ozturk S (2016). DNA methyltransferases exhibit dynamic expression during spermatogenesis. Reprod Biomed Online.

[102] Li N, Shen Q, Hua J (2016). Epigenetic Remodeling in Male Germline Development. Stem Cells Int.

[103] Yao C, Liu Y, Sun M, Niu M, Yuan Q, Hai Y (2015). MicroRNAs and DNA methylation as epigenetic regulators of mitosis, meiosis and spermiogenesis. Reproduction.

[104] Ooi SKT, Qiu C, Bernstein E, Li K, Jia D, Yang Z (2007). DNMT3L connects unmethylated lysine 4 of histone H3 to de novo methylation of DNA. Nature.

[105] Guo F, Li X, Liang D, Li T, Zhu P, Guo H (2014). Active and passive demethylation of male and female pronuclear DNA in the mammalian zygote. Cell Stem Cell.

[106] Wu X, Zhang Y (2017). TET-mediated active DNA demethylation: mechanism, function and beyond. Nat Rev Genet.

[107] Ehrlich M, Gama-Sosa MA, Huang LH, Midgett RM, Kuo KC, McCune RA (1982). Amount and distribution of 5-methylcytosine in human DNA from different types of tissues of cells. Nucleic Acids Res.

[108] Globisch D, Münzel M, Müller M, Michalakis S, Wagner M, Koch S (2010). Tissue distribution of 5-hydroxymethylcytosine and search for active demethylation intermediates. PLoS ONE.

[109] Ito S, D’Alessio AC, Taranova OV, Hong K, Sowers LC, Zhang Y (2010). Role of Tet proteins in 5mC to 5hmC conversion, ES–cell self–renewal and inner cell mass specification. Nature.

[110] Tahiliani M, Koh KP, Shen Y, Pastor WA, Bandukwala H, Brudno Y (2009). Conversion of 5–methylcytosine to 5–hydroxymethylcytosine in mammalian DNA by MLL partner TET1. Science.

[111] Caldwell BA, Bartolomei MS. DNA methylation reprogramming of genomic imprints in the mammalian germline: A TET-centric view. Andrology. 2022;in press: 10.1111/andr.13303 10.1111/andr.13303 36150101

[112] Yang J, Bashkenova N, Zang R, Huang X, Wang J (2020). The roles of TET family proteins in development and stem cells. Development..

[113] Cui X-L, Nie J, Ku J, Dougherty U, West-Szymanski DC, Collin F (2020). A human tissue map of 5-hydroxymethylcytosines exhibits tissue specificity through gene and enhancer modulation. Nat Commun.

[114] Ecsedi S, Rodriguez-Aguilera JR, Hernandez-Vargas H (2018). 5–hydroxymethylcytosine (5hmC), or how to identify your favorite cell. Epigenomes..

[115] Hackett JA, Sengupta R, Zylicz JJ, Murakami K, Lee C, Down TA (2013). Germline DNA demethylation dynamics and imprint erasure through 5-hydroxymethylcytosine. Science.

[116] Guz J, Gackowski D, Foksinski M, Rozalski R, Olinski R (2014). Comparison of the absolute level of epigenetic marks 5-methylcytosine, 5-hydroxymethylcytosine, and 5-hydroxymethyluracil between human leukocytes and sperm. Biol Reprod.

[117] Chioccarelli T, Pierantoni R, Manfrevola F, Porreca V, Fasano S, Chianese R (2020). Histone Post-Translational Modifications and CircRNAs in Mouse and Human Spermatozoa: Potential Epigenetic Marks to Assess Human Sperm Quality. J Clin Med.

[118] Godmann M, Auger V, Ferraroni-Aguiar V, Di Sauro A, Sette C, Behr R (2007). Dynamic regulation of histone H3 methylation at lysine 4 in mammalian spermatogenesis. Biol Reprod.

[119] Marmorstein R (2003). Structure of SET domain proteins: a new twist on histone methylation. Trends Biochem Sci.

[120] Yang Q, Yang Y, Zhou N, Tang K, Lau WB, Lau B (2018). Epigenetics in ovarian cancer: premise, properties, and perspectives. Mol Cancer.

[121] Collins RE, Tachibana M, Tamaru H, Smith KM, Jia D, Zhang X (2005). In vitro and in vivo analyses of a Phe/Tyr switch controlling product specificity of histone lysine methyltransferases. J Biol Chem.

[122] Zhai Q, Wang L, Zhao P, Li T (2017). Role of citrullination modification catalyzed by peptidylarginine deiminase 4 in gene transcriptional regulation. Acta Biochim Biophys Sin (Shanghai).

[123] Legube G, Trouche D (2003). Regulating histone acetyltransferases and deacetylases. EMBO Rep.

[124] Thiagarajan D, Vedantham S, Ananthakrishnan R, Schmidt AM, Ramasamy R (2016). Mechanisms of transcription factor acetylation and consequences in hearts. Biochim Biophys Acta.

[125] Milazzo G, Mercatelli D, Di Muzio G, Triboli L, De Rosa P, Perini G (2020). Histone Deacetylases (HDACs): Evolution, Specificity, Role in Transcriptional Complexes, and Pharmacological Actionability. Genes (Basel).

[126] Wang L, Xu Z, Khawar MB, Liu C, Li W (2017). The histone codes for meiosis. Reproduction.

[127] Oh E, Akopian D, Rape M (2018). Principles of Ubiquitin-Dependent Signaling. Annu Rev Cell Dev Biol.

[128] Mattiroli F, Penengo L (2021). Histone Ubiquitination: An Integrative Signaling Platform in Genome Stability. Trends Genet.

[129] Mulder MPC, Witting KF, Ovaa H (2020). Cracking the Ubiquitin Code: The Ubiquitin Toolbox. Curr Issues Mol Biol.

[130] Osley MA, Fleming AB, Kao C-F (2006). Histone ubiquitylation and the regulation of transcription. Results Probl Cell Differ.

[131] Liao Y, Sumara I, Pangou E (2022). Non-proteolytic ubiquitylation in cellular signaling and human disease. Commun Biol.

[132] Ntorla A, Burgoyne JR (2021). The regulation and function of histone crotonylation. Front Cell Dev Biol.

[133] Cavalieri V (2021). The Expanding Constellation of Histone Post-Translational Modifications in the Epigenetic Landscape. Genes (Basel).

[134] Wang T, Gao H, Li W, Liu C (2019). Essential role of histone replacement and modifications in male fertility. Front Genet.

[135] Talamilo A, Barroso-Gomila O, Giordano I, Ajuria L, Grillo M, Mayor U (2020). The role of SUMOylation during development. Biochem Soc Trans.

[136] Metzler-Guillemain C, Depetris D, Luciani JJ, Mignon-Ravix C, Mitchell MJ, Mattei M-G (2008). In human pachytene spermaotocytes, SUMO protein is restricted to the constitutive heterochromatin. Chromosome Res.

[137] Kekäläinen J, Hiltunen J, Jokiniemi A, Kuusipalo L, Heikura M, Leppänen J (2022). Female-induced selective modification of sperm protein SUMOylation-potential mechanistic insights into the non-random fertilization in humans. J Evol Biol.

[138] Marchiani S, Tamburrino L, Ricci B, Nosi D, Cambi M, Piomboni P (2014). SUMO1 in human sperm: new targets, role in motility and morphology and relationship with DNA damage. Reproduction.

[139] Vigodner M, Lucas B, Kemeny S, Schwartz T, Levy R (2020). Identification of sumoylated targets in proliferating mouse spermatogonia and human testicular seminomas. Asian J Androl.

[140] Hundertmark T, Gärtner SMK, Rathke C, Renkawitz-Pohl R (2018). Nejire/dCBP-mediated histone H3 acetylation during spermatogenesis is essential for male fertility in Drosophila melanogaster. PLoS ONE.

[141] Moretti C, Vaiman D, Tores F, Cocquet J (2016). Expression and epigenomic landscape of the sex chromosomes in mouse post-meiotic male germ cells. Epigenetics Chromatin.

[142] Dai L, Peng C, Montellier E, Lu Z, Chen Y, Ishii H (2014). Lysine 2-hydroxyisobutyrylation is a widely distributed active histone mark. Nat Chem Biol.

[143] Meyer-Ficca ML, Ihara M, Lonchar JD, Meistrich ML, Austin CA, Min W (2011). Poly(ADP-ribose) metabolism is essential for proper nucleoprotein exchange during mouse spermiogenesis. Biol Reprod.

[144] Meyer-Ficca ML, Ihara M, Bader JJ, Leu NA, Beneke S, Meyer RG (2015). Spermatid head elongation with normal nuclear shaping requires ADP-ribosyltransferase PARP11 (ARTD11) in mice. Biol Reprod.

[145] Guo F, Yan L, Guo H, Li L, Hu B, Zhao Y (2015). The Transcriptome and DNA Methylome Landscapes of Human Primordial Germ Cells. Cell.

[146] Ben Maamar M, Nilsson EE, Skinner MK (2021). Epigenetic transgenerational inheritance, gametogenesis and germline development. Biol Reprod.

[147] Ratajczak MZ, Zuba-Surma EK, Wysoczynski M, Ratajczak J, Kucia M (2008). Very small embryonic-like stem cells: characterization, developmental origin, and biological significance. Exp Hematol.

[148] Ratajczak MZ, Ratajczak J, Kucia M (2019). Very small embryonic-like stem cells (VSELs). Circ Res.

[149] Kaushik A, Bhartiya D (2022). Testicular cancer in mice: interplay between stem cells and endocrine insults. Stem Cell Res Ther.

[150] Kaushik A, Bhartiya D (2020). Additional Evidence to Establish Existence of Two Stem Cell Populations Including VSELs and SSCs in Adult Mouse Testes. Stem Cell Rev Rep.

[151] Ramathal C, Pera RR, Turek P. Embryonic Stem Cells and the Germ Cell Lineage: IntechOpen; 2011 2011/09/15/.

[152] Wen L, Tang F (2019). Human Germline Cell Development: from the Perspective of Single-Cell Sequencing. Mol Cell.

[153] Kurimoto K, Ikeda H, Kobayashi H. Chapter 1 - Epigenome reprogramming in the male and female germ line. In: Tollefsbol T, editor. Epigenetics and Reproductive Health. 21: Academic Press; 2021. p. 3–25.

[154] Sharma S, Wistuba J, Pock T, Schlatt S, Neuhaus N (2019). Spermatogonial stem cells: updates from specification to clinical relevance. Hum Reprod Update.

[155] Payne CJ (2013). Cycling to and from a stem cell niche: the temporal and spatial odyssey of mitotic male germ cells. Int J Dev Biol.

[156] Filipponi D, Feil R (2009). Perturbation of genomic imprinting in oligozoospermia. Epigenetics.

[157] Kubo N, Toh H, Shirane K, Shirakawa T, Kobayashi H, Sato T (2015). DNA methylation and gene expression dynamics during spermatogonial stem cell differentiation in the early postnatal mouse testis. BMC Genomics.

[158] Mochizuki K, Tachibana M, Saitou M, Tokitake Y, Matsui Y (2012). Implication of DNA demethylation and bivalent histone modification for selective gene regulation in mouse primordial germ cells. PLoS ONE.

[159] Kawabata Y, Kamio A, Jincho Y, Sakashita A, Takashima T, Kobayashi H (2019). Sex-specific histone modifications in mouse fetal and neonatal germ cells. Epigenomics.

[160] Ge S-Q, Lin S-L, Zhao Z-H, Sun Q-Y (2017). Epigenetic dynamics and interplay during spermatogenesis and embryogenesis: implications for male fertility and offspring health. Oncotarget.

[161] Maeda I, Okamura D, Tokitake Y, Ikeda M, Kawaguchi H, Mise N (2013). Max is a repressor of germ cell-related gene expression in mouse embryonic stem cells. Nat Commun.

[162] Matsui Y, Mochizuki K (2014). A current view of the epigenome in mouse primordial germ cells. Mol Reprod Dev.

[163] Eguizabal C, Herrera L, De Oñate L, Montserrat N, Hajkova P, Izpisua Belmonte JC (2016). Characterization of the epigenetic changes during human gonadal primordial germ cells reprogramming. Stem Cells.

[164] Chen H, Mruk D, Xiao X, Cheng CY, Winters SJ, Huhtaniemi IT (2017). Male Hypogonadism: Basic, Clinical and Therapeutic Principles. Contemporary Endocrinology. Human Spermatogenesis and Its Regulation.

[165] Sin H-S, Kartashov AV, Hasegawa K, Barski A, Namekawa SH (2015). Poised chromatin and bivalent domains facilitate the mitosis-to-meiosis transition in the male germline. BMC Biol.

[166] Song N, Liu J, An S, Nishino T, Hishikawa Y, Koji T (2011). Immunohistochemical Analysis of Histone H3 Modifications in Germ Cells during Mouse Spermatogenesis. Acta Histochem Cytochem.

[167] Payne C, Braun RE (2006). Histone lysine trimethylation exhibits a distinct perinuclear distribution in Plzf-expressing spermatogonia. Dev Biol.

[168] Ueda J, Harada A, Urahama T, Machida S, Maehara K, Hada M (2017). Testis-Specific Histone Variant H3t Gene Is Essential for Entry into Spermatogenesis. Cell Rep.

[169] Talbert PB, Henikoff S (2021). Histone variants at a glance. J Cell Sci..

[170] Ding D, Nguyen TT, Pang MYH, Ishibashi T (2021). Primate-specific histone variants. Genome.

[171] Shiraishi K, Shindo A, Harada A, Kurumizaka H, Kimura H, Ohkawa Y (2018). Roles of histone H3.5 in human spermatogenesis and spermatogenic disorders. Andrology..

[172] Urahama T, Harada A, Maehara K, Horikoshi N, Sato K, Sato Y (2016). Histone H3.5 forms an unstable nucleosome and accumulates around transcription start sites in human testis. Epigenet Chromatin..

[173] Maayan I (2012). Meiosis in Humans.

[174] L’Hernault SW, Maloy S, Hughes K (2013). Spermatocytes. Brenner's Encyclopedia of Genetics.

[175] Bennett MD (1977). The time and duration of meiosis. Philos Trans R Soc Lond B Biol Sci.

[176] Lam I, Keeney S (2014). Mechanism and regulation of meiotic recombination initiation. Cold Spring Harb Perspect Biol.

[177] Turner JMA (2007). Meiotic sex chromosome inactivation. Development.

[178] Fan X, Moustakas I, Torrens-Juaneda V, Lei Q, Hamer G, Louwe LA (2021). Transcriptional progression during meiotic prophase I reveals sex-specific features and X chromosome dynamics in human fetal female germline. PLoS Genet.

[179] Zuo W, Chen G, Gao Z, Li S, Chen Y, Huang C (2021). Stage-resolved Hi-C analyses reveal meiotic chromosome organizational features influencing homolog alignment. Nat Commun.

[180] Shirakata Y, Hiradate Y, Inoue H, Sato E, Tanemura K (2014). Histone h4 modification during mouse spermatogenesis. J Reprod Dev.

[181] Grey C, Baudat F, de Massy B (2018). PRDM9, a driver of the genetic map. PLoS Genet.

[182] Powers NR, Parvanov ED, Baker CL, Walker M, Petkov PM, Paigen K (2016). The Meiotic Recombination Activator PRDM9 Trimethylates Both H3K36 and H3K4 at Recombination Hotspots In Vivo. PLoS Genet.

[183] Zeng J, Yi SV (2014). Specific modifications of histone tails, but not DNA methylation, mirror the temporal variation of mammalian recombination hotspots. Genome Biol Evol.

[184] Keeney S (2008). Spo11 and the Formation of DNA Double-Strand Breaks in Meiosis. Genome Dyn Stab.

[185] Talibova G, Bilmez Y, Ozturk S. Increased double-strand breaks in aged mouse male germ cells may result from changed expression of the genes essential for homologous recombination or nonhomologous end joining repair. Histochem Cell Biol. 2022 Oct 15. 10.1007/s00418-022-02157-2. Online ahead of print. 10.1007/s00418-022-02157-2 36241856

[186] Nakamura K, Kato A, Kobayashi J, Yanagihara H, Sakamoto S, Oliveira DVNP (2011). Regulation of homologous recombination by RNF20-dependent H2B ubiquitination. Mol Cell.

[187] Wang Y, Zhu T, Li Q, Liu C, Han F, Chen M (2015). Prmt5 is required for germ cell survival during spermatogenesis in mice. Sci Rep.

[188] Khalil AM, Boyar FZ, Driscoll DJ (2004). Dynamic histone modifications mark sex chromosome inactivation and reactivation during mammalian spermatogenesis. Proc Natl Acad Sci U S A.

[189] Gong F, Miller KM (2013). Mammalian DNA repair: HATs and HDACs make their mark through histone acetylation. Mutat Res.

[190] Mattiroli F, Vissers JHA, van Dijk WJ, Ikpa P, Citterio E, Vermeulen W (2012). RNF168 ubiquitinates K13–15 on H2A/H2AX to drive DNA damage signaling. Cell.

[191] Alavattam KG, Maezawa S, Andreassen PR, Namekawa SH (2021). Meiotic sex chromosome inactivation and the XY body: a phase separation hypothesis. Cell Mol Life Sci.

[192] Ontoso D, Acosta I, van Leeuwen F, Freire R, San-Segundo PA (2013). Dot1-dependent histone H3K79 methylation promotes activation of the Mek1 meiotic checkpoint effector kinase by regulating the Hop1 adaptor. PLoS Genet.

[193] Dottermusch-Heidel C, Klaus ES, Gonzalez NH, Bhushan S, Meinhardt A, Bergmann M, Renkawitz-Pohl R, Rathke C, Steger K (2014). H3K79 methylation directly precedes the histone-to-protamine transition in mammalian spermatids and is sensitive to bacterial infections. Andrology.

[194] Nekrasov M, Soboleva TA, Jack C, Tremethick DJ (2013). Histone variant selectivity at the transcription start site: H2A.Z or H2A.Lap1. Nucleus..

[195] Turner JMA, Mahadevaiah SK, Ellis PJI, Mitchell MJ, Burgoyne PS (2006). Pachytene asynapsis drives meiotic sex chromosome inactivation and leads to substantial postmeiotic repression in spermatids. Dev Cell.

[196] Namekawa SH, Park PJ, Zhang L-F, Shima JE, McCarrey JR, Griswold MD (2006). Postmeiotic sex chromatin in the male germline of mice. Curr Biol.

[197] Soboleva TA, Parker BJ, Nekrasov M, Hart-Smith G, Tay YJ, Tng WQ (2017). A new link between transcriptional initiation and pre-mRNA splicing: The RNA binding histone variant H2A.B. PLoS Genet..

[198] Kohestani H, Wereszczynski J (2021). Effects of H2A.B incorporation on nucleosome structures and dynamics. Biophys J..

[199] Eberlin A, Grauffel C, Oulad-Abdelghani M, Robert F, Torres-Padilla M-E, Lambrot R (2008). Histone H3 tails containing dimethylated lysine and adjacent phosphorylated serine modifications adopt a specific conformation during mitosis and meiosis. Mol Cell Biol.

[200] Kim S, Namekawa SH, Niswander LM, Ward JO, Lee JT, Bardwell VJ (2007). A mammal-specific Doublesex homolog associates with male sex chromatin and is required for male meiosis. PLoS Genet.

[201] O'Donnell L (2014). Mechanisms of spermiogenesis and spermiation and how they are disturbed. Spermatogenesis.

[202] de la Iglesia A, Jodar M, Oliva R, Castillo J. Insights into the sperm chromatin and implications for male infertility from a protein perspective. WIREs Mech Dis. 2022;e1588. 10.1002/wsbm.1588. Online ahead of print. 10.1002/wsbm.1588 36181449

[203] Balhorn R (2007). The protamine family of sperm nuclear proteins. Genome Biol.

[204] Rathke C, Baarends WM, Awe S, Renkawitz-Pohl R (2014). Chromatin dynamics during spermiogenesis. Biochim Biophys Acta.

[205] Arevalo L, Merges GE, Schneider S, Oben FE, Neumann IS, Schorle H (2022). Loss of the cleaved-protamine 2 domain leads to incomplete histone-to-protamine exchange and infertility in mice. PLoS Genet..

[206] Hamad MF (2019). Quantification of histones and protamines mRNA tran–scripts in sperms of infertile couples and their impact on sperm’s quality and chromatin integrity. Reprod Biol.

[207] Okada Y (2022). Sperm chromatin structure: Insights from in vitro to in situ experiments. Curr Opin Cell Biol.

[208] Bao J, Bedford MT (2016). Epigenetic regulation of the histone-to-protamine transition during spermiogenesis. Reproduction.

[209] Ketchum CC, Larsen CD, McNeil A, Meyer-Ficca ML, Meyer RG (2018). Early histone H4 acetylation during chromatin remodeling in equine spermatogenesis. Biol Reprod.

[210] Wendt KD, Shilatifard A (2006). Packing for the germy: the role of histone H4 Ser1 phosphorylation in chromatin compaction and germ cell development. Genes Dev.

[211] Krishnamoorthy T, Chen X, Govin J, Cheung WL, Dorsey J, Schindler K (2006). Phosphorylation of histone H4 Ser1 regulates sporulation in yeast and is conserved in fly and mouse spermatogenesis. Genes Dev.

[212] Laberge R-M, Boissonneault G (2005). On the nature and origin of DNA strand breaks in elongating spermatids. Biol Reprod.

[213] Wang X, Kang J-Y, Wei L, Yang X, Sun H, Yang S (2019). PHF7 is a novel histone H2A E3 ligase prior to histone-to-protamine exchange during spermiogenesis. Development..

[214] Her YR, Wang L, Chepelev I, Manterola M, Berkovits B, Cui K (2021). Genome-wide chromatin occupancy of BRDT and gene expression analysis suggest transcriptional partners and specific epigenetic landscapes that regulate gene expression during spermatogenesis. Mol Reprod Dev.

[215] Manterola M, Brown TM, Oh MY, Garyn C, Gonzalez BJ, Wolgemuth DJ (2018). BRDT is an essential epigenetic regulator for proper chromatin organization, silencing of sex chromosomes and crossover formation in male meiosis. PLoS Genet.

[216] Kumar A, Raut S, Balasinor NH (2018). Endocrine regulation of sperm release. Reprod Fertil Dev.

[217] Barrachina F, Battistone MA, Castillo J, Mallofré C, Jodar M, Breton S (2022). Sperm acquire epididymis-derived proteins through epididymosomes. Hum Reprod.

[218] Sullivan R, Mieusset R (2016). The human epididymis: its function in sperm maturation. Hum Reprod Update.

[219] Chen H, Scott-Boyer M-P, Droit A, Robert C, Belleannee (2022). Sperm Heterogeneity Accounts for Sperm DNA Methylation Variations Observed in the Caput Epididymis, Independently From DNMT/TET Activities. Front Cell Dev Biol..

[220] Park Y-J, Lee B-M, Pang W-K, Ryu D-Y, Rahman MS, Pang MG (2022). Low Sperm Motility Is Determined by Abnormal Protein Modification during Epididymal Maturation. World J Mens Health.

[221] Godmann M, Zemter S, Kosan C (2017). Genetic and epigenetic mouse models of human male infertility. In: Genetics of Human Infertiity. Monogr Hum Genet..

[222] Hanna CW, Demond H, Kelsey G (2018). Epigenetic regulation in development: is the mouse a good model for the human?. Hum Reprod Update.

[223] Bedi YS, Roach AN, Thomas KN, Mehta NA, Golding MC (2022). Chromatin alterations during the epididymal maturation of mouse sperm refine the paternally inherited epigenome. Epigenet Chromatin.

[224] Pepin A-S, Lafleur C, Lambrot R, Dumeaux V, Kimmins S (2022). Sperm histone H3 lysine 4 tri-methylation serves as a metabolic sensor of paternal obesity and is associated with the inheritance of metabolic dysfunction. Mol Metab.

[225] Marcho C, Oluwayiose OA, Pilsner JR (2020). The preconception environment and sperm epigenetics. Andrology.

[226] Rodriguez JB, Sanchez CC (2019). Epigenetic Transgenerational Inheritance. Adv Exp Med Biol.

[227] Di Persio S, Leitão E, Wöste M, Tekath T, Cremers JF, Dugas M (2021). Whole-genome methylation analysis of testicular germ cells from cryptozoospermic men points to recurrent and functionally relevant DNA methylation changes. Clin Epigenet.

